# Targeting KIF23 inhibits cell proliferation and primary chemoresistance in cervical cancer by inactivating the MYH9/MCM2/PCNA pathway

**DOI:** 10.1002/ctm2.70652

**Published:** 2026-04-06

**Authors:** Ying Zhu, Qian Wang, Yilin Zhang, Yahui Liu, Haini Fu, Zike Yang, Xiaojie Deng, Suiqun Guo

**Affiliations:** ^1^ Department of Obstetrics and Gynecology The Third Affiliated Hospital, Southern Medical University Guangzhou China; ^2^ School of Basic Medical Sciences Guangdong Medical University Dongguan China; ^3^ Department of Oncology Zhongshan Hospital of Xiamen University, School of Medicine, Xiamen University Xiamen China; ^4^ Department of Pulmonary and Critical Care Medicine The Second Affiliated Hospital of Guangzhou Medical University Guangzhou China

**Keywords:** cervical cancer, chemoresistance, KIF23

## Abstract

**Background:**

Kinesin family member 23 (KIF23) is recognised as an important tumour promoter involved in the pathogenesis of various cancers. However, its role and underlying molecular mechanisms in regulating cervical cancer (CC) growth and primary chemoresistance remain to be fully elucidated.

**Methods:**

The expression and prognostic significance of KIF23 were initially assessed through bioinformatic analyses and subsequently validated in clinical specimens. To evaluate the effects of KIF23 on cell proliferation and cisplatin (DDP) sensitivity in CC cells, in vitro and in vivo experiments were conducted using CRISPR/Cas9 knockout, overexpression and mouse xenograft models. Co‐immunoprecipitation, protein half‐life assays and ubiquitination assays were employed to elucidate the interactions and regulatory mechanisms involving KIF23, myosin heavy chain 9 (MYH9), minichromosome maintenance protein 2 (MCM2) and proliferating cell nuclear antigen (PCNA), thereby revealing the molecular basis of KIF23‐mediated CC progression and primary chemoresistance.

**Results:**

KIF23 is highly expressed in CC tissues and is significantly correlated with poor prognosis and DDP resistance in patients. The knockout of KIF23 inhibited cell proliferation, induced G1‐phase arrest and enhanced chemosensitivity to DDP. Mechanistically, the C‐terminal domain of KIF23 was found to directly bind to the myosin tail domain of MYH9. This interaction stabilises MYH9 by recruiting deubiquitinase 7 (ubiquitin‐specific protease 7 [USP7]), which removes K48‐linked ubiquitin chains. The consequent upregulation of MYH9 promoted the recruitment of ubiquitin‐specific protease 15 (USP15) to deubiquitinate MCM2, thereby preventing its degradation. Lysine 469 (K469) of MCM2 was identified as the key site for MYH9‐induced deubiquitination. Furthermore, elevated MCM2 levels enhanced its binding to PCNA, thereby promoting CC cell proliferation.

**Conclusions:**

These findings demonstrated that elevated KIF23 levels act as an unfavorable prognostic factor for CC by promoting cell proliferation and primary chemoresistance via the activation of the MYH9/MCM2/PCNA axis. Thus, KIF23 may represent a promising therapeutic target for improving clinical outcomes in CC.

**Highlights:**

- Cisplatin treatment induces KIF23 expression in a concentration‐ and time‐dependent manner.
- KIF23 recruits USP7, which removes the K48‐linked ubiquitin chain of MYH9, thereby stabilising MYH9 and facilitating its nuclear transport.
- MYH9 recruits USP15, thereby stabilising MCM2, which, in turn, regulates the G1/S phase transition by binding to PCNA.
- Targeting the KIF23/MYH9/MCM2/PCNA axis sensitises cervical cancer cells to cisplatin.

## Graphical abstract

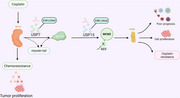

## 1 INTRODUCTION

Cervical cancer (CC) continues to be a major contributor to cancer‐associated deaths in women across the globe.^1^, ^2^ Although the widespread administration of human papillomavirus vaccines and advances in early screening techniques have reduced the incidence to some extent, the prognosis for patients with advanced or recurrent CC remains poor.^3^, ^4^, ^5^, ^6^ Currently, cisplatin (DDP)‐based concurrent chemoradiotherapy is the standard first‐line treatment for locally advanced CC.^7^, ^8^, ^9^ However, the development of chemoresistance greatly limits the clinical efficacy of DDP, leading to treatment failure and tumour recurrence.^10^, ^11^, ^12^ Unlike acquired resistance induced during treatment, primary resistance frequently arises from intrinsic genetic features of tumour cells or the pre‑activation of specific oncogenes, rendering tumours inherently resistant upon initial exposure to chemotherapeutic drugs.^4^, ^13^ Therefore, elucidating the molecular mechanisms underlying primary chemoresistance and defining new targets for intervention are essential for improving CC treatment.

Kinesins constitute a family of motor proteins that facilitate cargo transport along microtubules via ATP hydrolysis. These proteins have a well‐established role in carcinogenesis and chemoresistance.^14^ CC remains a leading Kinesin family member 23 (KIF23) is a vital member of the kinesin family, playing a role in cell proliferation, migration and invasion.^15^, ^16^, ^17^ KIF23 has been implicated in the development of numerous malignancies, including endometrial,^18^ gastric,^19^ breast^20^ and colorectal cancers.^21^ However, its expression profile, clinical significance and association with primary chemoresistance in CC remain unclear.

Here, we found that high KIF23 expression is an unfavorable factor strongly linked to clinical progression, poor prognosis and chemotherapy resistance in CC. KIF23 binds to the tail region of myosin heavy chain 9 (MYH9) via its C‐terminal domain and recruits the deubiquitinase ubiquitin‐specific protease 7 (USP7), leading to MYH9 protein stabilisation. The resulting MYH9 upregulation further promotes the recruitment of ubiquitin‐specific peptidase 15 (USP15), which enhances the deubiquitination of minichromosome maintenance protein 2 (MCM2), thereby prolonging its half‐life.

These events ultimately lead to the strengthening of the interaction between MCM2 and proliferating cell nuclear antigen (PCNA), conferring stronger proliferative capacity to tumour cells and driving primary chemoresistance.

Our data reveal a new mechanism by which KIF23 induces cell growth and primary chemoresistance, providing a strong theoretical basis and potential intervention targets for overcoming DDP resistance in CC.

## 2 MATERIALS AND METHODS

### 2.1 Aberrant expression and prognostic role of KIF23

Differential expression of KIF23 was assessed using harmonised and normalised datasets obtained from the UCSC database.^22^ Expression data for KIF23 were obtained by multi‐sample extraction. In addition, gene set enrichment was executed via the Linkedomics^23^ platform. Finally, survival curves were plotted using the Kaplan–Meier method, where expression thresholds were delineated based on optimal cut‐point selection to differentiate between high‐ and low‐expression groups.

### 2.2 Cells and samples

Human CC cell lines SIHA and C33A were purchased from the Cell Bank of the Chinese Academy of Sciences, while the HEK293T cells were provided by the Cancer Research Institute, Southern Medical University. Prior to experimentation, all cell lines were validated via short tandem repeat profiling and routinely screened to ensure the absence of mycoplasma. All cells were incubated in Dulbecco's modified Eagle's medium (PM150210; Pricella) supplemented with 10% bovine serum (Nobimplex) in a humidified incubator at 37°C with 5% CO_2_. HEK293T cells were cultured under identical conditions. The study protocol was approved by the institutional ethics committee, and written informed consent was obtained from all patients before tissue collection.

### 2.3 Transfection

CC cells were transfected with plasmid constructs encoding USP7 (Miaoling Biology), MYH9 (Vigene Biosciences), MCM2 (Miaoling Biology) and USP15 (Miaoling Biology) using Lipofectamine 3000 (model L2000‐015; Invitrogen) as per the manufacturer's protocols. Transfection was performed when the cells reached 40% confluence. For targeted gene silencing, specific short interfering RNAs (siRNAs) targeting MCM2 were obtained from RiboBio Inc. Recombinant plasmids carrying mutant MCM2 constructs were provided by Miaoling Biology.

### 2.4 Lentivirus infection

CRISPR/Cas9‐mediated *KIF23* knockout (KO) lentiviral constructs targeting distinct coding regions of KIF23 (designated as KO‐KIF23‐1# and KO‐KIF23‐2#) were generated by Guangzhou Aiji Biotechnology Co., Ltd. Cells were transfected with lentiviruses and then screened for green fluorescent protein positivity to identify successfully transduced cells. Short hairpin RNAs (shRNAs) targeting MYH9 were designed based on the reference sequence NM_002473.5 and cloned into the pLVTHM‐GFP lentiviral vector (Table S1). Knockdown efficiency was confirmed by reverse transcription‐quantitative polymerase chain reaction (RT‐qPCR) and Western blotting (WB) assays.

### 2.5 RT‐qPCR

Intracellular RNA was isolated utilising an extraction kit (Foregene) and subsequently converted into complementary DNA employing a specialised reverse transcription kit (Vazyme). Quantitative real‐time PCR amplification was performed using a CFX96 real‐time PCR system (Bio‐Rad). The sequences of the primers used in this study are listed in Table S2.

### 2.6 WB analysis

Whole‐cell lysates were prepared using lysis buffer, and the protein content was assessed by a BCA assay (Beijing Tiangen Biotechnology Co., Ltd.). Equal quantities of protein extracts were separated via electrophoresis and transferred to PVDF membranes. Following blocking, the membranes were incubated overnight with primary antibodies, and then probed with corresponding secondary antibodies. Comprehensive information about the primary antibodies used is provided in Table S3.

### 2.7 Molecular docking

Three‐dimensional atomic coordinates of the proteins of interest were retrieved from the RCSB database (https://www.rcsb.org).^24^ For structure‐based docking, the cryo‐electron microscopy structure of KIF23 (PDB: 4ETO) was designated as the receptor, with the X‐ray crystallography structure of MYH9 (PDB: 3VHX) serving as the ligand. In subsequent docking simulations, MYH9 (PDB: 3VHX) was used as the receptor for the MCM2 ligand (PDB: 5C3I), which, in turn, served as the receptor for the PCNA ligand (PDB: 2HIK). AutoDock Vina (v1.2.0)^25^ was employed to carry out molecular docking and the derived binding interfaces were visualised with Discovery Studio 2019.

### 2.8 5‐Ethynyl‐2′‐deoxyuridine binding assay

Active cellular proliferation was quantified using a commercial 5‐ethynyl‐2′‐deoxyuridine (EdU) incorporation kit (Cell‐Light Apollo567). In brief, after a 2‐h incubation with 50 µM EdU, cells were fixed in 4% paraformaldehyde followed by permeabilisation with.5% Triton X‐100. Incorporated EdU was labelled with a fluorescent Apollo probe, and nuclei were counterstained with DAPI (C1005).

### 2.9 Clone formation assay

To assess long‐term proliferative capacity, approximately 500 cells were seeded into individual wells of six‐well plates. After 14 days of culture, the visible colonies underwent fixation, crystal violet staining and manual enumeration.

### 2.10 Co‐immunoprecipitation assay

Cells were disrupted using a specialised IP Lysis Buffer (87787; Thermo Scientific) supplemented with a cocktail of protease inhibitors, and then cellular homogenates were incubated overnight under chilled conditions (4°C) with target‐specific antibodies or non‐specific control immunoglobulins. Antigen‒antibody complexes were subsequently retrieved through incubation with protein A/G magnetic beads (B23202; Bimake). After three rounds of washing buffer washes, the samples were heated to denaturation in SDS loading buffer and subjected to WB analysis.

### 2.11 Immunofluorescence staining

CC cells harbouring the specified plasmids were plated onto sterile glass coverslips. After fixation in 4% paraformaldehyde for 30 min, cells were permeabilised with.2% Triton X‐100 and incubated with primary antibodies overnight. Bound antibodies were detected using the corresponding secondary antibodies, and nuclear counterstaining was performed using DAPI (C1005; Beyotime Biotechnology).

### 2.12 Nuclear and cytoplasmic extraction tests

To analyse cytoplasmic and nuclear proteins separately, subcellular fractions were isolated using a fractionation kit (Thermo Fisher Scientific) as per the manufacturer's protocol. All procedures were performed at 4°C or on ice. Intact cells were chemically lysed and centrifuged to obtain the soluble cytoplasmic fraction. The remaining nuclear pellets were resuspended in a dedicated extraction buffer to release nuclear proteins. The fractions were quantified and stored for downstream analyses.

### 2.13 Cycloheximide tracking assay

To evaluate protein turnover, CC cells were exposed to cycloheximide (CHX; S7418; Selleck) for the indicated durations, and target protein degradation was examined by WB. For proteasome inhibition, cells were treated with 20 µM MG132 (S2619; Selleck). Protein lysates were prepared using RIPA lysis buffer supplemented with SDS loading buffer, followed by immunoblot analysis with anti‐MYH9 or anti‐MCM2 antibodies. For immunoprecipitation, protein samples were denatured at 95°C for 10 min prior to antibody incubation.

### 2.14 in vivo tumourigenesis in nude mice

To validate the impact of KIF23 on tumour growth and drug sensitivity within in vivo, we established a subcutaneous xenograft model using juvenile (4‐week‐old) athymic BALB/c mice. Suspensions of CC cells in the logarithmic growth phase were prepared from stably transfected cells, including those from the KO‐KIF23, overexpression KIF23 (oe‐KIF23) and oe‐KIF23 + short hairpin (sh)‐MYH9 (KIF23 overexpression combined with shRNA‐mediated MYH9 knockdown) groups. Seven days after cell injection, each mouse in each group received an intraperitoneal injection every 3 days, with DDP (4 mg/kg) or phosphate‐buffered saline administered. Based on this, the KO‐KIF23 + DDP, oe‐KIF23 + DDP and oe‐KIF23 + sh‐MYH9 + DDP combined treatment groups were established.

This dosing regimen was based on a prior safety assessment, and no significant systemic toxicity or abnormal weight loss was observed in the mice during the experiment. Tumour volumes were monitored every 3 days with a Vernier caliper. After 28 days, the BALB/c nude mice were humanely sacrificed, and tumour tissues were harvested for subsequent histological examination. All in vivo experimental procedures were performed in strict accordance with the institutional animal care and use guidelines.

### 2.15 Immunohistochemistry

Immunohistochemistry (IHC) was performed to assess the expression of KIF23, MYH9, MCM2, Ki67 and PCNA following the kit's recommended protocols. The experimental design adhered to a previously described methodology.^26^ A dual‐scoring system was used for microscopic evaluation, according to staining intensity and the proportion of positive cells. Samples with a combined score of ≥ 6 were classified as exhibiting high expression.

### 2.16 Statistical analyses

Statistical analyses were performed using GraphPad Prism 8 (GraphPad Software). Differences between two groups were assessed using an independent two‐tailed *t*‐test, while comparisons among multiple groups were conducted using one‐way analysis of variance. A *p*‐value < .05 was regarded as statistically significant.

## 3 RESULTS

### 3.1 KIF23 is highly expressed in CC and is associated with poor prognosis and primary chemoresistance

**FIGURE 1:**
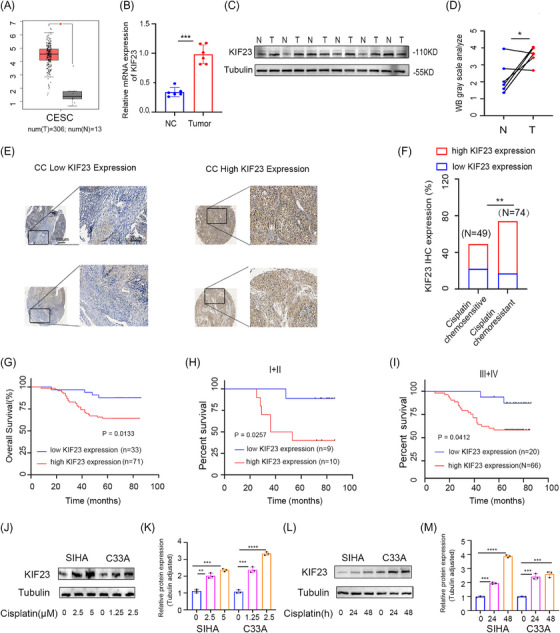
High expression of kinesin family member 23 (KIF23) promotes cervical cancer (CC) progression and is associated with a poorer prognosis. (A) Analysis of differential KIF23 expression in CC tissues using the Gene Expression Profiling Interactive Analysis (GEPIA) database. (B) Expression levels of KIF23 mRNA in normal tissues and CC tissues. (C and D) Detection of KIF23 expression in CC tissues and adjacent normal tissues (N) via Western blotting (WB) analysis. (E and F) Immunohistochemistry (IHC) staining analysis of KIF23 in clinical samples (cisplatin [DDP]‐resistant group, *n* = 74; DDP‐sensitive group, *n* = 49). (G) Kaplan–Meier survival analysis showing that high KIF23 expression is associated with a poorer overall survival rate in 104 patients with CC. (H and I) Stratified survival analysis of KIF23 expression in CC patients with clinical stages I–II and III–IV. (J and K) DDP induces KIF23 protein expression in a dose‐dependent manner in CC cells. (L and M) DDP upregulates KIF23 expression in a time‐dependent manner. Statistical significance was determined by Student's *t*‐test (^*^ *p* < 0.05, ^**^ *p* < 0.01, ^***^ *p* < 0.001). Magnification: 40× (scale bar: 100 µm); 200× (scale bar: 20 µm).

DDP‐based chemotherapy remains the primary treatment for CC; however, the development of drug resistance and subsequent recurrence often result in a poor prognosis.^27^, ^28^ DDP is known to exert anti‐tumour effects predominantly by inducing DNA damage and triggering cell cycle arrest. Meanwhile, KIF23—a critical regulator of cell division—has been suggested to contribute to chemoresistance and is associated with proliferation and treatment sensitivity in various cancers.^14^, ^29^ To evaluate the potential role of KIF23 in CC, its expression levels were analysed using data from the UALCAN database. We observed a marked upregulation of KIF23 in CC tissues (Figure S1A). Furthermore, prognostic evaluation and survival analysis indicated that high KIF23 expression was associated with poorer overall survival and progression‐free survival (Figure S1B–F). To support these bioinformatic findings, data from The Cancer Genome Atlas (TCGA) database were evaluated, revealing that KIF23 mRNA levels are significantly elevated in CC cell lines (Figure 1A). To further validate the expression of KIF23 in CC, we examined its levels in six fresh CC specimens and adjacent tissues using RT‐qPCR and WB. Our findings demonstrated that KIF23 expression was substantially elevated in CC tissues relative to paired adjacent non‐tumour tissues (Figure 1B,D).

To further investigate its clinical significance and association with chemotherapeutic response, immunohistochemical staining was performed on clinical tissue sections. Our results demonstrated that KIF23 expression was higher in CC samples that showed chemoresistance to DDP than in those that showed chemosensitivity to the drug (Figure 1E,F and Table 1). Survival analysis indicated that CC patients with low KIF23 expression exhibited longer overall survival than those with high KIF23 expression (*p* = 0.0133, Figure 1G). In addition, among patients stratified into stage I–II (*p* = 0.0257) and stage III–IV (*p* = 0.0412) groups, elevated KIF23 expression was associated with significantly poorer overall survival (Figure 1H,I and Table S4). Further multivariate Cox regression analysis confirmed that high KIF23 expression serves as an independent adverse prognostic indicator in CC patients (Table S5).

**TABLE 1:** Correlation between kinesin family member 23 (KIF23) expression and sensitivity to cisplatin (DDP) in cervical cancer (CC) (*χ* ^2^‐test).

		Expression of KIF23		
Group	Case (*n*)	Low expression	High expression	*p*‐value
DDP chemosensitive	49	22 (44.9%)	27 (55.1%)	< 0.05
DDP chemoresistant	74	17 (23%)	57 (77%)	

Given the close association between KIF23 expression and both DDP primary chemoresistance and adverse prognosis, we hypothesised that DDP treatment itself may influence KIF23 expression levels. Subsequent in vitro assays confirmed that DDP treatment significantly increased KIF23 expression in CC cells in both concentration‐ and time‐dependent manners (Figure 1J–M). These results implied that high KIF23 expression in CC patients correlates with a poorer prognosis and may promote DDP‐based chemoresistance.

### 3.2 Knockout of KIF23 inhibited the proliferation of CC cells and enhanced their sensitivity to DDP

**FIGURE 2:**
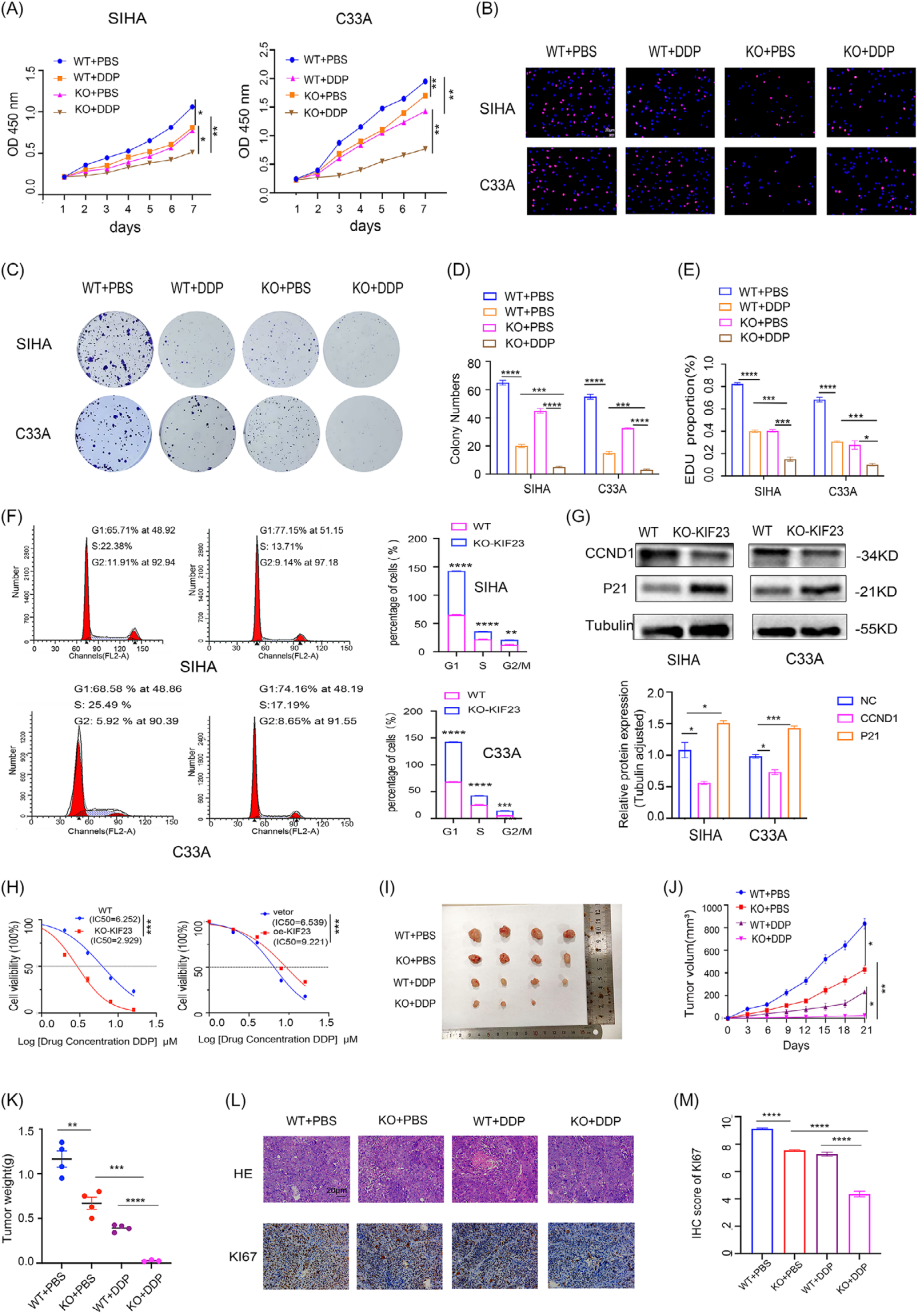
Effects of KIF23 on the proliferation and cell cycle of CC cells. (A) Cell Counting Kit‐8 (CCK‐8) assay showing that knockout of KIF23 significantly inhibits cell viability. Data are presented as mean ± SD. (Student's *t*‐test: ^*^ *p* < 0.05, ^**^ *p* < 0.01). (B) 5‐Ethynyl‐2′‐deoxyuridine (EdU) incorporation assay in CC cells with or without *KIF23* knockout (KO) (scale bar: 20 µm). (C‒E) Colony formation assay in CC cells with or without *KIF23* KO. (F) Flow cytometry analysis showing that *KIF23* KO induces G1 phase cell cycle arrest. (G) Western blotting (WB) analysis of CCND1 and P21 protein levels following *KIF23* KO. (H) IC50 values of SIHA and C33A cells, showing that *KIF23* KO significantly enhances sensitivity to DDP. (I) in vivo effects of KIF23 and DDP evaluated in a xenograft model derived from SIHA cells (*n* = 4 per group). (J and K) Analysis of tumour weight and volume for each group (wild‐type [WT], KO‐KIF23, WT + DDP and KO + DDP). (L) Representative haematoxylin and eosin (H&E) staining and immunohistochemistry (IHC) images of primary tumour tissues. (M) Quantitative analysis of Ki67 staining in subcutaneous tumours from nude mice. Results are shown as mean ± SD (Student's *t*‐test: ^*^ *p* < 0.05, ^**^ *p* < 0.01, ^***^ *p *< 0.001).

To further explore the biological roles of KIF23 in CC, we knocked out the *KIF23* gene in SIHA and C33A cell lines through transfection (KO‐KIF23‐1# and KO‐KIF23‐2#). The transfection efficiency was evaluated using fluorescence labelling (Figure S2A) while KIF23 efficiency was assessed by RT‐qPCR. KO‐KIF23‐1# was noted to be the most effective at reducing KIF23 expression at both the mRNA (Figure S2B) and protein (Figure S2C) levels, and was therefore used for subsequent experiments. We next performed Cell Counting Kit‐8 (CCK‐8), EdU and colony formation assays and obtained consistent results. Knocking out KIF23 delayed the proliferation and colony formation of CC cells. Notably, knocking out KIF23 further strengthened the inhibitory effect of DDP on cell proliferation (Figure 2A–E).

To elucidate the molecular mechanisms underlying KIF23 function in CC, we performed KEGG pathway enrichment analysis. The results indicated that KIF23 is primarily involved in cell cycle‐related biological processes (Figure S2D). Flow cytometric analysis revealed that knocking out KIF23 led to CC cell arrest in the G1 phase (Figure 2F). WB analysis further indicated that the deletion of KIF23 in CC cells downregulated the expression of CCND1 and upregulated that of P21 (Figure 2G), indicating that KIF23 influences cell proliferation via the regulation of the G1/S transition. Additionally, IC50 experiment results showed that the KO of KIF23 significantly reduced the primary resistance of CC cells to DDP, while its overexpression increased the IC50 value (Figures 2H and S2E). These findings confirmed that KIF23 negatively regulates the chemosensitivity of CC cells.

To investigate the in vivo function of KIF23, we established a subcutaneous xenograft mouse model (Figure 2I). Tumour growth and weight were both lower in the KO‐KIF23 + DDP group relative to the control group (Figure 2J,K). Additionally, IHC assays demonstrated that Ki67 expression levels were lower in both the KO‐KIF23 and KO‐KIF23 + DDP groups compared with the control group (Figure 2L,M). These findings suggested that knocking out KIF23 can significantly inhibit the proliferation of CC cells and enhance their chemosensitivity.

### 3.3 KIF23 and MYH9 interact with USP7

Given the crucial role of KIF23 in cell division and proliferation, we hypothesised that it may influence cell proliferation and drug resistance by regulating proteins related to these processes. MYH9, the heavy chain of non‐muscle myosin IIA, plays an important role in cytoskeletal, proliferative and cell cycle‐associated dynamics, and its dysregulated expression is closely associated with the occurrence and progression of various tumours and chemoresistance.^30^, ^31^ These observations suggested that MYH9 may be an important KIF23 downstream effector molecule.

**FIGURE 3:**
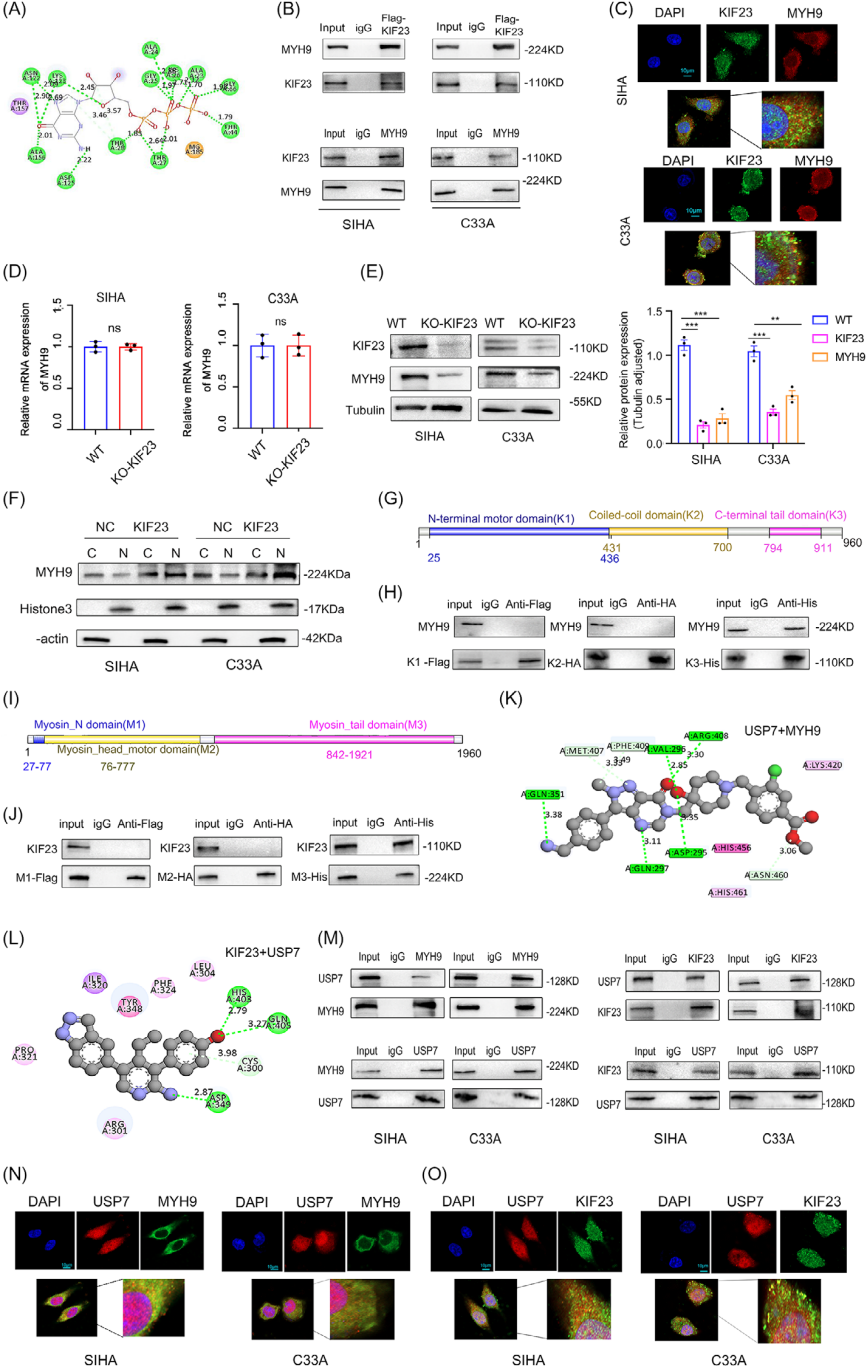
KIF23 and myosin heavy chain 9 (MYH9) interact with ubiquitin‐specific protease 7 (USP7). (A) Molecular docking results for KIF23 and MYH9. (B) Co‐immunoprecipitation (Co‐IP) analysis to detect the interaction between KIF23 and MYH9 in CC cells. (C) Immunofluorescence (IF) analysis showing the co‐localisation of KIF23 and MYH9 in CC cells (scale bar: 10 µm). (D) Reverse transcription‐quantitative polymerase chain reaction (RT‐qPCR) analysis of *MYH9* mRNA levels in CC cells with or without *KIF23* KO. (E) Western blotting (WB) analysis of MYH9 protein levels in CC cells with or without *KIF23* KO. (F) Nuclear and cytoplasmic fractionation assay to detect MYH9 protein levels in overexpression *KIF23* (oe‐*KIF23*) CC cells. (G) Schematic diagram of the functional domains of KIF23. (H) Co‐IP assay demonstrating the interaction between different KIF23 domains and MYH9 in HEK‐293T cells transfected with the corresponding constructs. (I) Schematic diagram of the functional domains of MYH9. (J) Co‐IP assay demonstrating the interaction between different MYH9 domains and KIF23 in HEK‐293T cells. (K and L) Molecular docking diagrams of MYH9 with USP7 and KIF23 with USP7. (M) Co‐IP analysis in CC cells demonstrating the interactions of MYH9 and KIF23 with USP7. (N and O) Representative confocal images showing the co‐localisation of MYH9 with USP7 and KIF23 with USP7 (scale bar: 10 µm).

To investigate the mechanism by which KIF23 exerts its role in CC cells, we first used data from the BioGRID database (https://thebiogrid.org) and molecular docking simulations to predict the candidate proteins that potentially interact with KIF23 and MYH9 (Figure 3A and Table 2). Subsequently, co‐immunoprecipitation (Co‐IP) experiments confirmed that KIF23 interacts with MYH9 in CC cells (Figure 3B), while immunofluorescence analysis revealed that KIF23 and MYH9 co‐localised in the cytoplasm (Figure 3C). Because KIF23 interacts with MYH9, we sought to determine whether KIF23 is involved in the regulation of MYH9 expression. Interestingly, WB results showed that the KO of KIF23 reduced MYH9 protein expression, whereas the mRNA levels of *MYH9* remained unchanged (Figure 3D,E). Nuclear–cytoplasmic fractionation experiments revealed that KIF23 promotes the nuclear transport of MYH9 (Figure 3F). Additionally, domain analysis revealed that the C‐terminal tail domain of KIF23 (K3) binds to the Myosin_tail domain of MYH9 (M3), establishing the structural basis for their interaction (Figure 3G–J).

**TABLE 2:** Predicted interactor for KIF23 in BioGRID.

Interactor	Organism	Description	Evidence
MYH9	Homo sapiens	Myosin, heavy chain 9, non‐muscle	1

Next, we investigated whether KIF23 can recruit binding partners, such as deubiquitinating enzymes, to stabilise MYH9. Based on BioGRID analysis, we identified USP7 as a candidate MYH9‐ and KIF23‐binding protein (Table 3). Molecular docking and Co‐IP experiments confirmed that USP7 interacts with both MYH9 and KIF23 (Figure 3K–M). In line with this, confocal microscopy further demonstrated that USP7 co‐localised with both MYH9 and KIF23 in the cytoplasm of CC cells (Figure 3N,O). In summary, these findings suggested that both KIF23 and MYH9 interact with USP7.

**TABLE 3:** Predicted interactors for ubiquitin‐specific protease 7 (USP7) in BioGRID.

Interactor	Organism	Description	Evidence
KIF23	Homo sapiens	Kinesin family member 23	2
MYH9	Homo sapiens	Myosin, heavy chain 9, non‐muscle	1

### 3.4 KIF23 induced the K48‐linked deubiquitination of MYH9 by recruiting USP7

**FIGURE 4:**
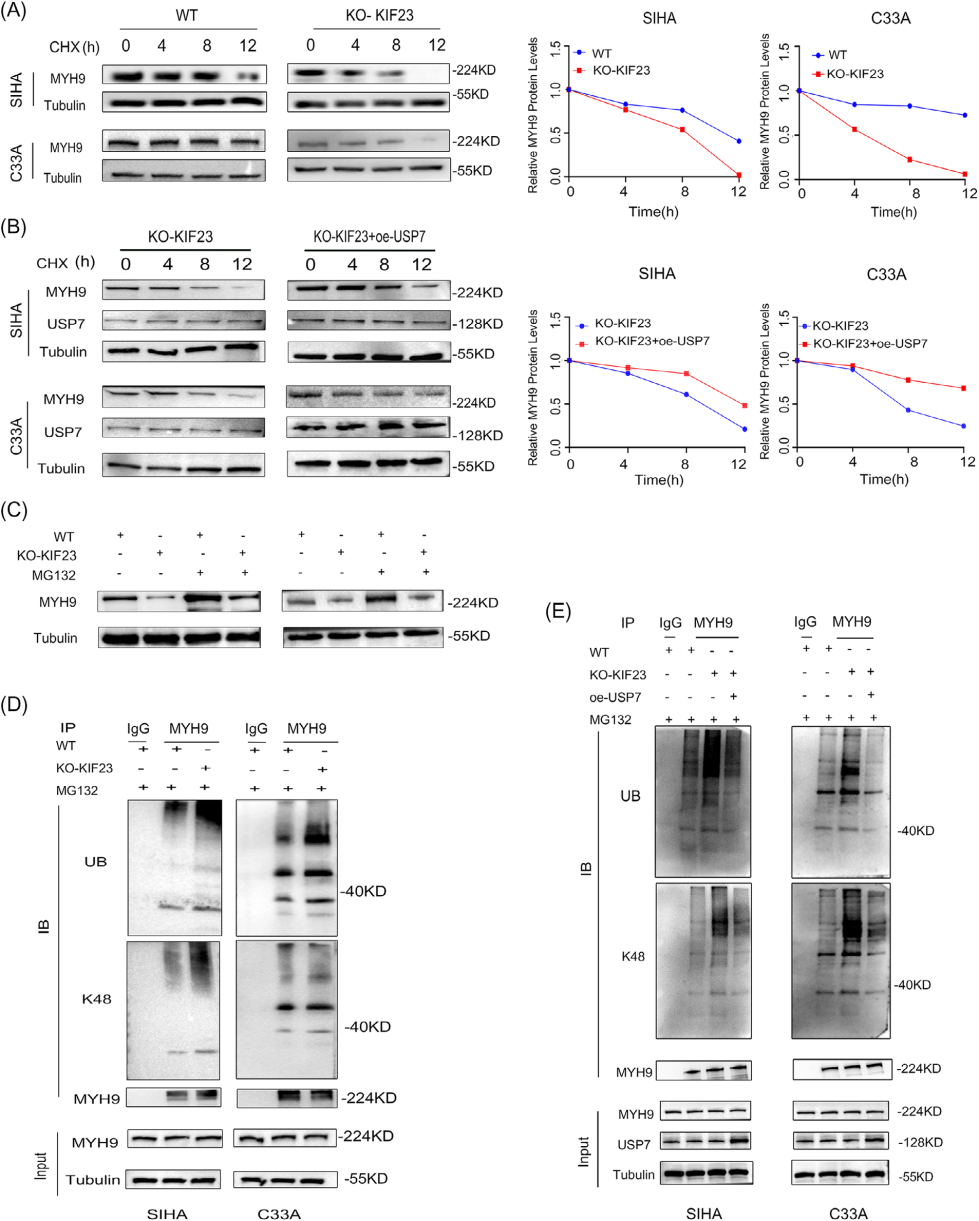
KIF23 induces K48‐linked deubiquitination of MYH9 through recruitment of USP7. (A) Western blotting (WB) analysis of MYH9 protein stability at different time points following cycloheximide (CHX) treatment in control and *KIF23* KO groups. (B) CHX chase assay examining the regulation of MYH9 protein stability by USP7 overexpression (oe‐*USP7*). (C) Effect of MG132 treatment (12 h) on MYH9 protein stability in *KIF23* KO CC cells and their controls. (D) Co‐IP and WB analyses of the effect of *KIF23* KO on MYH9 ubiquitination levels after 12 h of MG132 treatment. (E) Co‐IP and WB assays assessing the effects of control, *KIF23* KO and *KIF23* KO combined with USP7 overexpression (KO‐*KIF23* + oe‐*USP7*) on MYH9 ubiquitination after 12 h of MG132 treatment in SIHA (left) and C33A (right) cells.

Subsequently, we investigated whether KIF23 stabilises MYH9 protein by recruiting USP7. We found that the overexpression of USP7 significantly prolonged the half‐life of MYH9 protein under KIF23 KO conditions, an effect that was effectively antagonised by USP7 overexpression (Figure 4A,B). Conversely, the overexpression of KIF23 prolonged the half‐life of MYH9, whereas the knockdown of USP7 promoted its degradation (Figure S3A–C). Notably, treatment with MG132 for 12 h reversed the MYH9 downregulation induced by KIF23 KO, and the overexpression of KIF23 reversed the KIF23‐mediated downregulation of MYH9 (Figures 4C and S3D). WB results showed that KIF23 KO reduced the K48‐linked deubiquitination of MYH9 in a USP7‐dependent manner (Figure 4D,E). Importantly, ubiquitination analysis confirmed that in the presence of USP7, the overexpression of KIF23 increased K48‐linked deubiquitination levels in MYH9 (Figure S3E,F). These results supported our initial hypothesis, namely, that KIF23 induces K48‐related deubiquitination of MYH9 through the recruitment of USP7, thereby stabilising the MYH9 protein.

### 3.5 MCM2 interacts with USP15 and MYH9

**FIGURE 5:**
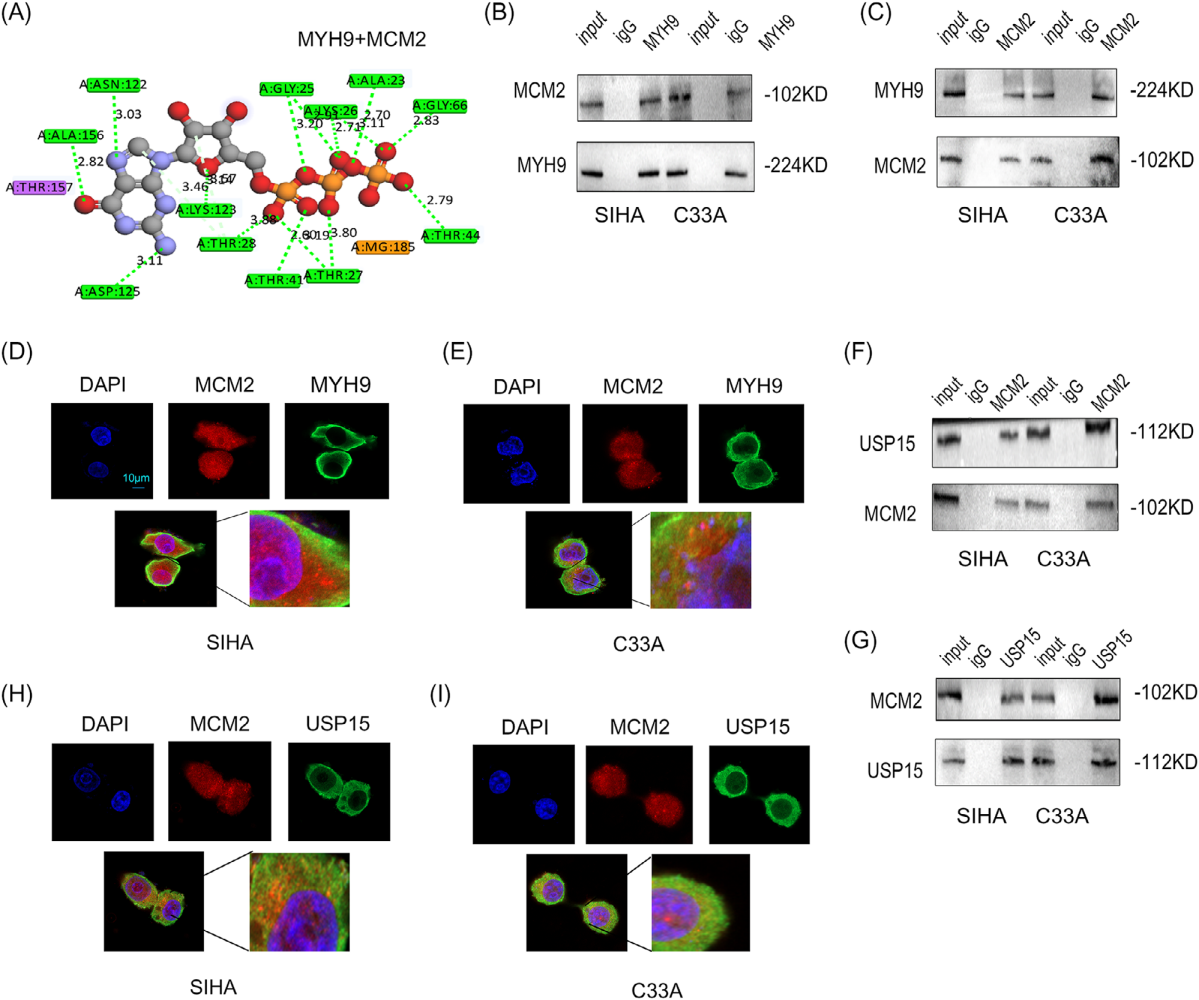
Ubiquitin‐specific protease 15 (USP15) and MYH9 interact with minichromosome maintenance protein 2 (MCM2). (A) Molecular docking predicting the interaction between MYH9 and MCM2. (B and C) Co‐IP assay showing the interaction between MYH9 and MCM2 in CC cells. (D and E) Confocal microscopy visualising the co‐localisation of MYH9 and MCM2 in CC cells. (F and G) Co‐IP assay examining the interaction between USP15 and MCM2 in CC cells. (H and I) Confocal microscopy visualising the co‐localisation of MCM2 and USP15 in CC cells. Scale bar: 10 µm.

Considering the central role of MYH9 in cell proliferation, cytoskeletal remodelling and cell cycle regulation, we hypothesised that MYH9 might promote proliferation and chemoresistance by regulating proteins essential for the initiation of DNA replication. MCM2 is a critical protein in this process, and its aberrant expression is frequently associated with tumour progression. BioGRID predictions and preliminary molecular docking confirmed that MYH9 and MCM2 directly interact (Figure 5A). Moreover, USP15 was identified as a potential binding partner of MCM2 (Table 4). Accordingly, we next sought to determine whether MYH9 mediates the deubiquitination of MCM2 via USP15. The results showed that MCM2 interacts with both MYH9 and USP15 (Figure 5B,C,F,G), and immunofluorescence analysis further revealed that these proteins co‐localise in the nuclei of CC cells (Figure 5D,E,H,I). The above data indicated that MCM2 interacts and co‐localises with MYH9 and USP15 in CC cells.

**TABLE 4:** Predicted interactors for ubiquitin‐specific protease 15 (USP15) in BioGRID.

Interactor	Organism	Description	Evidence
MCM2	Homo sapiens	Minichromosome maintenance complex component 2	1

### 3.6 MYH9 and USP15 cooperatively inhibited the ubiquitination‐mediated degradation of MCM2 and promoted its nuclear translocation

**FIGURE 6:**
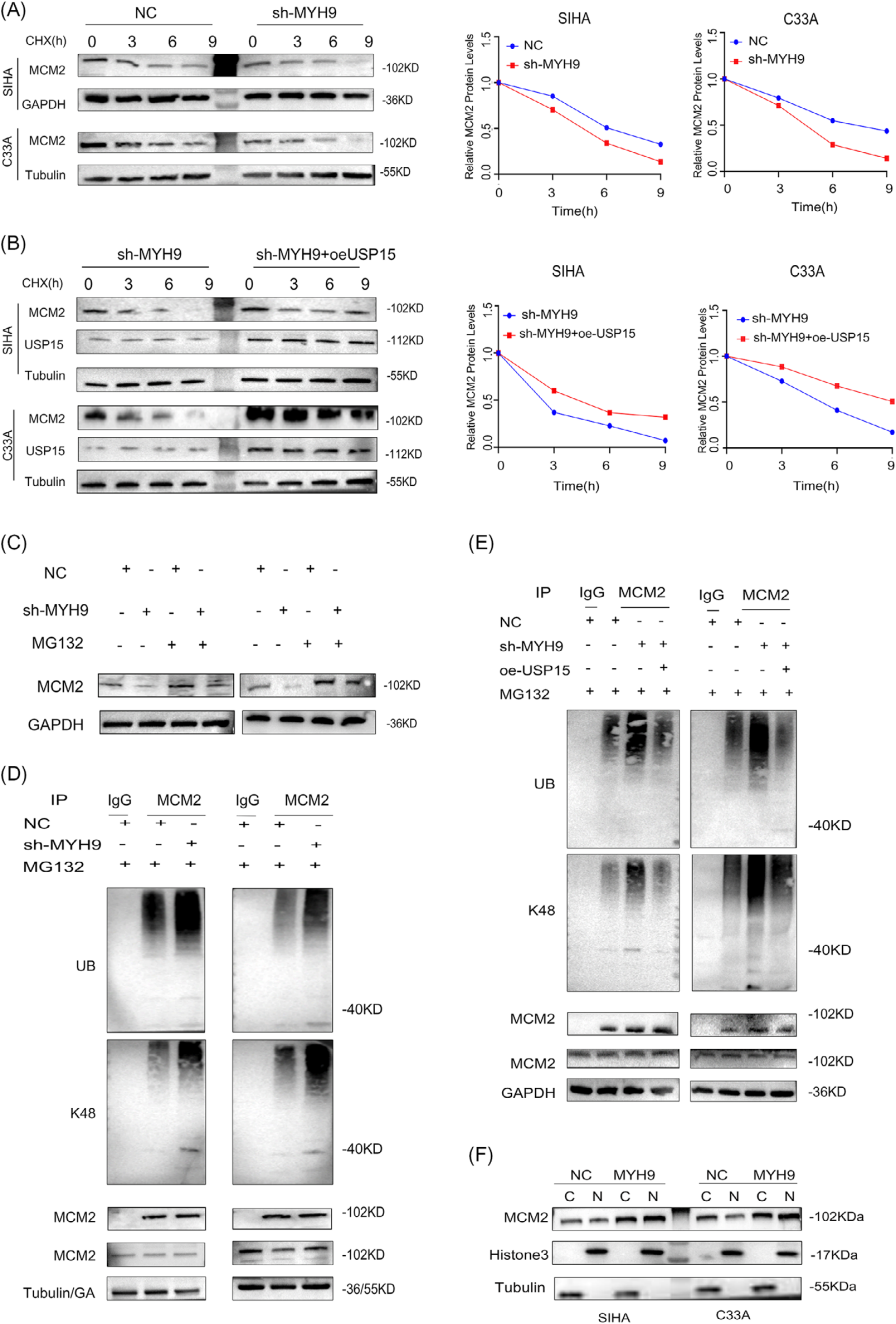
MYH9 knockdown suppresses K48‐linked deubiquitination of MCM2 through USP15 recruitment. (A) CHX chase assay assessing the half‐life of MCM2 protein in short hairpin (sh)‐*MYH9* and control CC cells. (B) CHX chase assay examining the regulation of MCM2 protein stability by overexpression USP15 (oe‐USP15). (C) Effect of MG132 treatment (9 h) on MCM2 protein stability in *MYH9* knockdown and control CC cells. (D) Co‐IP and WB analyses of the effect of *MYH9* knockdown on MCM2 ubiquitination levels after 9 h of MG132 treatment. (E) Co‐IP and WB analyses of the effects of *MYH9* knockdown alone or combined with USP15 overexpression on MCM2 ubiquitination levels in CC cells following 9 h of MG132 treatment in SIHA (left) and C33A (right) cells. (F) Nuclear and cytoplasmic fractionation experiments showing that MYH9 overexpression increases MCM2 protein levels in the nucleus.

**FIGURE 7:**
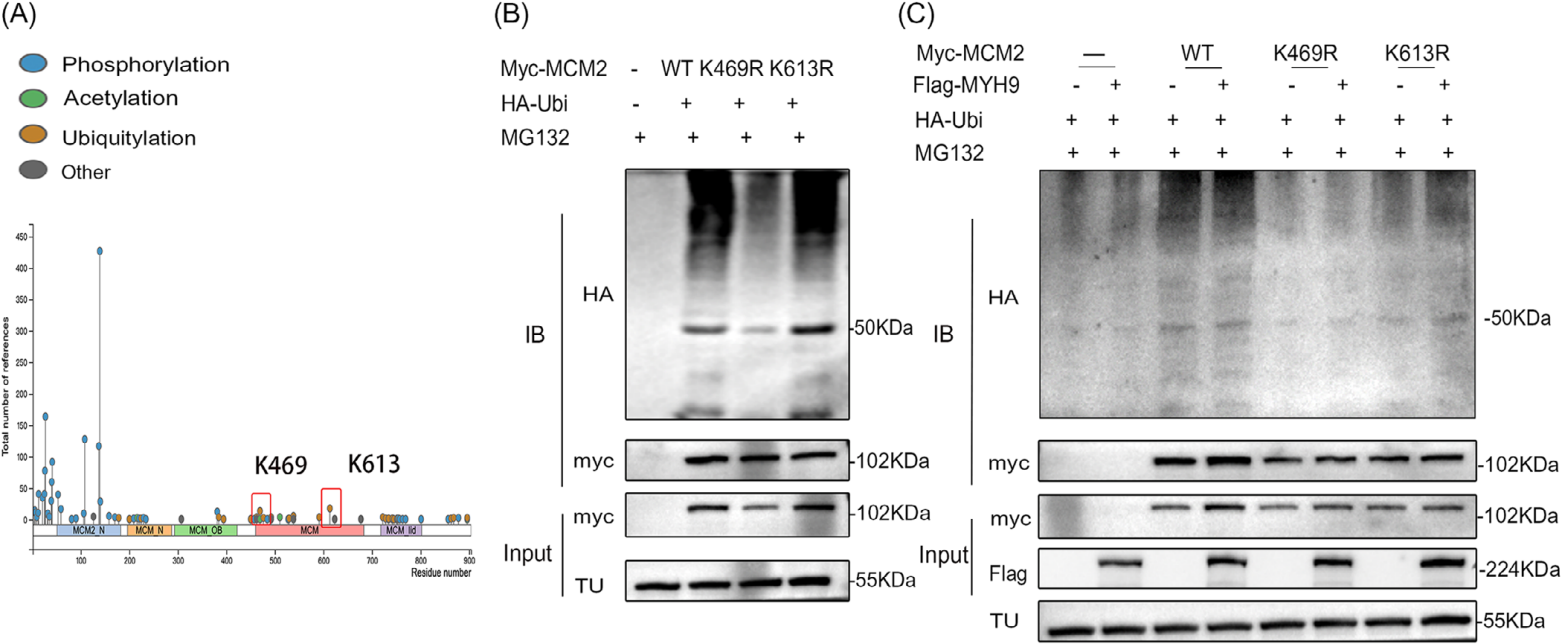
Predicted binding sites between MCM2 and MYH9. (A) Identification of potential ubiquitination sites on MCM2. (B) Comparison of ubiquitination levels between wild‐type MCM2 and MCM2 with various mutations. (C) Analysis of MCM2 ubiquitination at the K469R and K613R mutant sites in the presence of MYH9.

To further investigate whether MYH9 affects MCM2 expression, stability assays were performed using CHX. The findings revealed that the knockdown of MYH9 accelerated MCM2 protein degradation (Figure 6A); however, the overexpression of USP15 (oe‐USP15) partially restored the stability of MCM2 following MYH9 knockdown (Figure 6B). Conversely, MYH9 overexpression inhibited the degradation of endogenous MCM2, while the knockdown of USP15 (si‐USP15) shortened the half‐life of MCM2 protein (Figure S4A,B). The downregulation of MCM2 induced by MYH9 knockdown was reversed by treatment with the proteasome inhibitor MG132 (Figure 6C). Moreover, the overexpression of MYH9 reversed the MCM2 downregulation resulting from MYH9 knockdown (Figure S4C). Further experiments confirmed that the knockdown of MYH9 inhibited the USP15‐mediated removal of MCM2 K48‐linked ubiquitin chains (Figure 7D,E), thereby promoting its degradation. Importantly, ubiquitination analysis confirmed that in the presence of USP15, the overexpression of MYH9 increased the K48‐linked deubiquitination of MCM2 (Figure S4D,E). Additionally, analysis of nuclear and cytoplasmic extracts showed that MYH9 overexpression increased the protein levels of MCM2 in the nucleus but decreased them the cytoplasm (Figure 6F). In summary, these results implied that MYH9 can stabilise MCM2 protein and promote its nuclear translocation, thereby enhancing chemoresistance.

### 3.7 Lysine 469 (K469) is a key regulatory site for the MYH9‐induced ubiquitination modification of MCM2

To further elucidate the mechanism involved in the MYH9‐mediated regulation of MCM2, potential ubiquitination sites were predicted at lysine residues K469 and K613 using data from the PhosphoSitePlus and GPS‐UBER databases (Figure 7A and Table 5). These lysine residues were separately substituted with arginine to block the ubiquitination process, and these two mutant forms of MCM2, along with wild‐type MCM2, were subsequently transfected into HEK‐293T cells. We observed that the K469 mutation significantly reduced the ubiquitination of MCM2 (Figure 7B), demonstrating that K469 is a key functional site responsible for regulating MCM2 stability (Figure 7C).

**TABLE 5:** NCBI reference sequence: NP_004517.2 minichromosome maintenance protein 2 (MCM2) homo sapiens (http://gpsuber.biocuckoo.cn/wsresult.php).

Position	Code	E3 enzyme	Peptide	Score	Cutoff
178	K	General	IESIENLEDLKGHSVREWVSM	0.6132	0.3566
216	K	General	HVDSHGHNVFKERISDMCKEN	0.4239	0.3566
224	K	General	VFKERISDMCKENRESLVVNY	0.5202	0.3566
384	K	General	QRIRIQESPGKVAAGRLPRSK	0.6390	0.3566
**469**	K	General	EDVKMITSLSKDQQIGEKIFA	**0.7475**	**0.3** **566**
505	K	General	LALALFGGEPKNPGGKHKVRG	0.3663	0.3566
529	K	General	VLLCGDPGTAKSQFLKYIEKV	0.5769	0.3566
591	K	General	RGVCLIDEFDKMNDQDRTSIH	0.6710	0.3566
721	K	General	GVEPLPQEVLKKYIIYAKERV	0.6228	0.3566

Bold values shows the statistically significant results of K469.

### 3.8 MCM2 interacts with PCNA

Subsequently, we investigated the effect of MCM2 on PCNA expression in CC cells. We found that overexpression of MCM2 increased the protein expression of PCNA (Figure S5A), whereas its knockdown exerted the opposite effect (Figure S5B). Furthermore, BioGRID analysis and Co‐IP experiments confirmed that MCM2 interacted with PCNA (Figure S5C). Additionally, immunofluorescence assays showed that MCM2 and PCNA mainly co‐localised in nuclei of CC cells, with limited distribution in the cytoplasm (Figure S5D). These findings suggested that MCM2 interacts with PCNA and may influence its stability.

### 3.9 MYH9 is required for both the KIF23‐mediated proliferation of CC cells and their primary resistance to DDP

**FIGURE 8:**
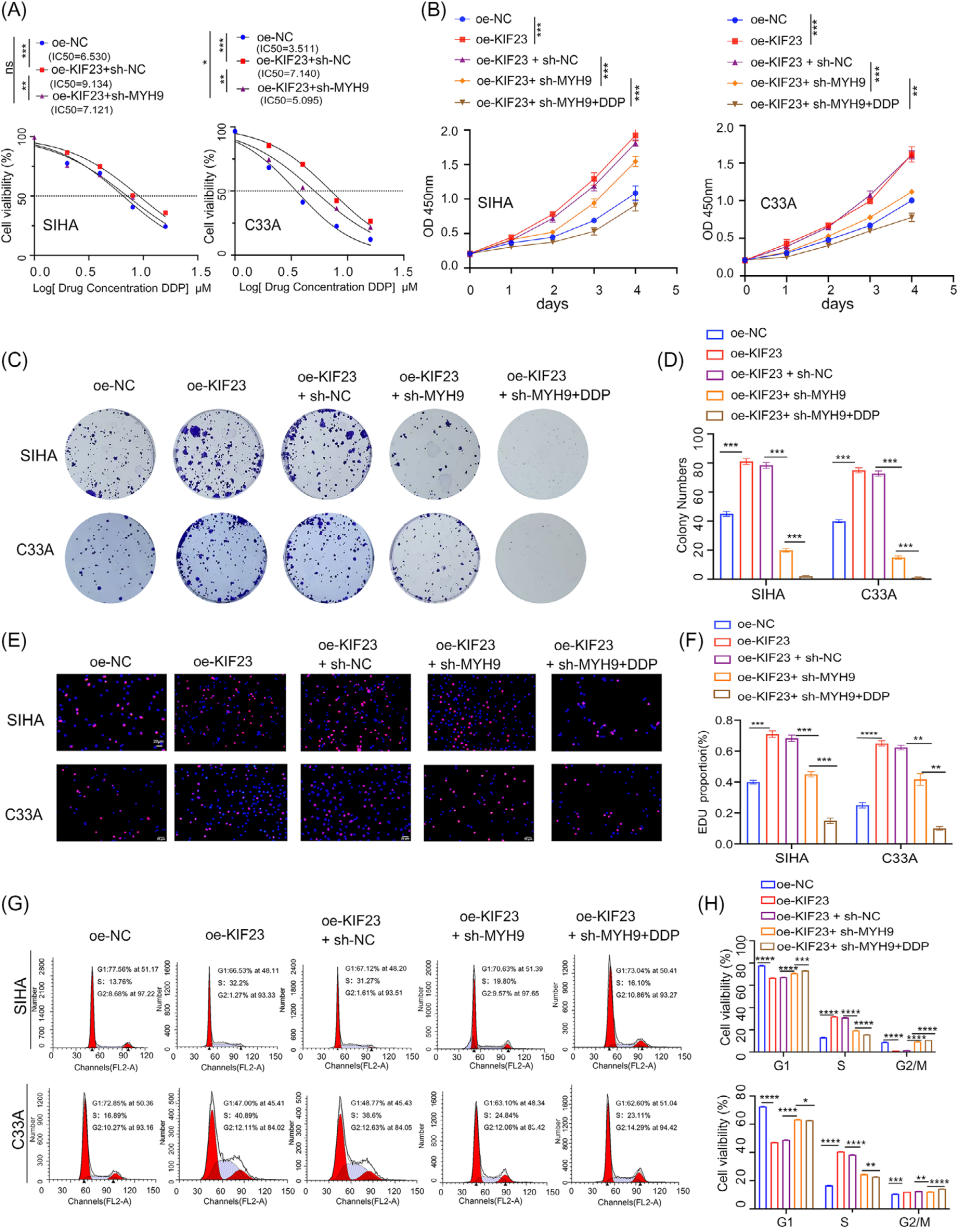
MYH9 is an essential downstream effector of KIF23 in promoting CC cell proliferation and DDP resistance. (A) Effect of DDP treatment on the survival rate of CC cells, evaluated by IC50 assay in overexpression *KIF23* oe‐*KIF23* cells, oe‐*KIF23* cells with short hairpin (sh)‐*MYH9* and their respective controls. (B) Cell Counting Kit‐8 (CCK‐8) assay determining the effect of *MYH9* knockdown on the proliferation of oe‐*KIF23* CC cells. (C) Colony formation assays examining the effect of *MYH9* knockdown on the proliferation of oe‐*KIF23* CC cells. (D) Quantification of the colony formation assay results. (E) EdU staining assessing the proliferation of CC cells with oe‐*KIF23* alone or in combination with *MYH9* knockdown, with or without DDP treatment. (F) Quantification of EdU‐positive cells. (G) Cell cycle distribution analysed by flow cytometry in CC cells with oe‐*KIF23* alone or in combination with *MYH9* knockdown, with or without DDP treatment. (H) Quantification of the percentage of cells in each cell cycle phase.

Next, we sought to clarify whether MYH9 plays a key role in the KIF23‐mediated malignant progression and primary DDP resistance in CC using both in vitro and in vivo experiments. The in vitro IC50 assay showed that the overexpression of the *KIF23* gene enhanced the resistance of CC cells to DDP, and this effect was effectively reversed when MYH9 was knocked down (Figure 8A). Subsequent CCK‐8, colony formation and EdU assays yielded consistent results, indicating that KIF23 overexpression significantly promotes CC cell proliferation. Furthermore, knocking down MYH9 effectively reversed the promotive effect of KIF23 overexpression on cell proliferation. Notably, this inhibition of proliferation was further potentiated following combined treatment with DDP (Figure 8B–F).

**FIGURE 9:**
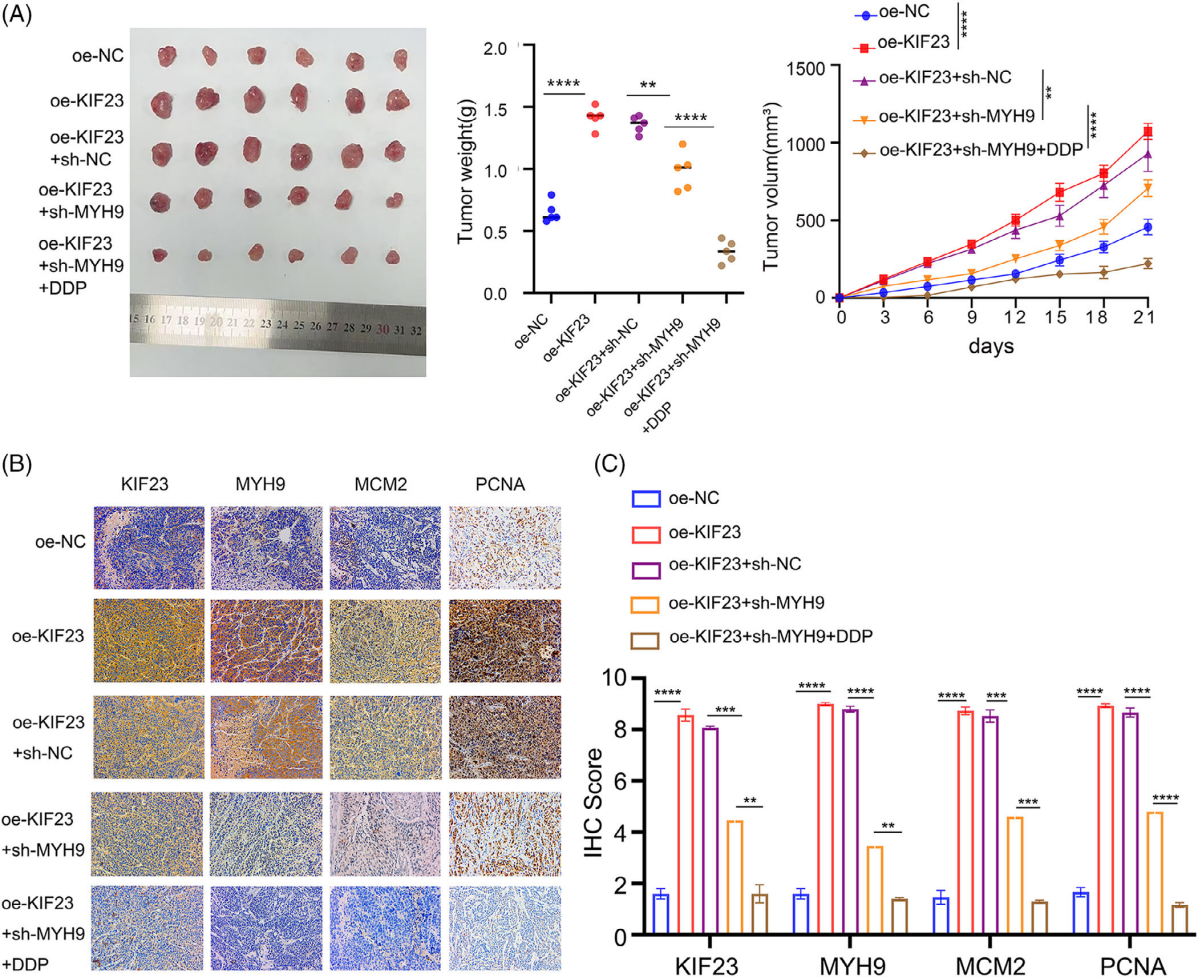
Crucial role of MYH9 in KIF23‐mediated tumourigenesis and DDP resistance in vivo. (A) Xenograft tumour models in nude mice. Tumour volume growth curves and tumour weight quantification for each group (*n* = 6 per group). (B) IHC detection of KIF23, MYH9, MCM2 and proliferating cell nuclear antigen (PCNA) expression levels. (C) Quantitative analysis of KIF23, MYH9, MCM2 and PCNA expression based on IHC results. Statistical significance was determined by Student's *t*‐test (^*^ *p* < 0.05, ^**^ *p* < 0.01). Scale bar: 20 µm.

Flow cytometric results additionally showed that the overexpression of KIF23 promoted the transition of CC cells from the G1 to the S phase of the cell cycle, while the knockdown of MYH9 and treatment with DDP reversed this effect, resulting in the inhibition of cell cycle progression and, consequently, cell proliferation (Figure 9G,H).

Finally, we validated the above findings in vivo using a mouse subcutaneous tumour xenograft model. The data revealed that both the tumour growth rate and final weight were significantly lower in the MYH9‐knockdown group relative to controls. Furthermore, the combined treatment (oe‐KIF23 + sh‐MYH9 + DDP) yielded the most substantial inhibition, resulting in the lowest tumour volumes and weights among all the groups (Figure 9A). Immunohistochemical analysis demonstrated that MYH9 knockdown effectively reversed the pro‐proliferative effect of KIF23, and this inhibitory effect was further enhanced upon combination with DDP chemotherapy (Figure 9B). These findings were further verified in the transplanted tumour tissues by WB (Figure 9C). Collectively, these data support the conclusion that MYH9 serves as a critical downstream mediator through which KIF23 promotes CC cell proliferation and primary DDP resistance.

## 4 DISCUSSION

In the study, we systematically demonstrated the pivotal function of KIF23 in promoting malignant progression and modulating the response to DDP in CC. Initial analyses of KIF23 expression via the UALCAN and TCGA databases indicated that KIF23 is significantly upregulated in various solid tumours, including CC. Subsequent survival analysis confirmed that elevated KIF23 expression positively correlates with advanced clinical stages and shorter overall survival. Furthermore, we also observed that KIF23 protein levels were upregulated in tumour samples from CC patients who received DDP‐based chemotherapy. These data are consistent with findings reported in other tumour types.^32^ Reinforcing the characterisation of KIF23 as a putative oncogene involved in the mediation of primary DDP resistance in CC. Furthermore, we found that DDP treatment induced the upregulation of KIF23 expression in both a time‐ and concentration‐dependent manner. This suggests that KIF23 functions not only as a driver of oncogenesis in CC but also as a stress‐responsive mediator of primary chemoresistance. Subsequent functional assays corroborated the oncogenic role of KIF23. Notably, we found that it markedly enhanced cell proliferation, accelerated G1/S phase progression and augmented primary resistance to DDP. These observations align with previous investigations into KIF23 in other tumour types,^14^ indicating that KIF23 is a critical molecule responsible for both tumour progression and the induction of DDP resistance in CC.

To elucidate the molecular pathways through which KIF23 drives CC progression and chemoresistance, candidate interacting partners and potential effectors of KIF23 were screened and validated. MYH9 is recognised as a tumour‐initiating factor that promotes cell proliferation and accelerates development in specific cancers.^33^, ^34^ Studies have shown that MYH9 is highly expressed in multiple malignancies, including lung cance,^35^ gastric cancer,^33^ hepatocellular carcinoma^36^, ^37^ and ovarian cancer,^38^ facilitating tumour expansion and augmenting chemoresistance.^39^

Analysis of the BioGRID database predicted a potential interaction between KIF23 and MYH9. Subsequent experimental validation confirmed that the C‐terminal domain of KIF23 binds to the myosin tail domain of MYH9. Immunofluorescence analysis further revealed that these two proteins exhibit significant co‐localisation in the cytoplasm. Furthermore, we found that KIF23 did not alter MYH9 mRNA levels but markedly increased MYH9 protein expression. These data suggest that KIF23 regulates MYH9 expression at the post‐translational modification level.

Mechanistic investigations revealed that KIF23 stabilises the MYH9 protein by recruiting the deubiquitinase USP7. USP7 is widely reported to regulate the ubiquitination levels of multiple proteins involved in tumourigenesis and therapeutic responses,^40^, ^41^, ^42^ and serves as a potential binding partner for both KIF23 and MYH9. We therefore hypothesised that KIF23 may positively regulate MYH9 expression by recruiting USP7, which mediates the MYH9 protein deubiquitination. To validate this hypothesis, protein half‐life assays and MG132 treatment experiments were performed. The results showed that KIF23 depletion accelerated MYH9 protein degradation, accompanied by an increase in K48‐linked polyubiquitination. In contrast, the overexpression of USP7 partially restored MYH9 stability. These data support the hypothesis that KIF23 recruits USP7 to remove K48‐linked ubiquitin chains from MYH9, thereby prolonging the protein half‐life and upregulating MYH9 expression.

To investigate the molecular mechanism underlying KIF23‑mediated regulation of MYH9‑driven malignant proliferation and chemoresistance in CC, we screened for downstream interacting partners of MYH9 and identified MCM2 via the BioGRID database. MCM2 is a core component of the DNA replication initiation complex, and its abnormal stability is closely associated with malignant transformation.^43^, ^44^ Studies have shown that elevated MCM2 expression is closely related to malignant transformation in ovarian and lung cancer.^45^, ^46^ In the present study, we confirmed that MYH9 significantly upregulates MCM2 protein levels and promotes its nuclear enrichment, but has only limited effects on MCM2 mRNA levels. These data indicate that MYH9 regulates MCM2 protein stability in a manner analogous to the regulation of MYH9 by KIF23. Based on BioGRID database predictions and experimental validation, we found that USP15 may form a complex with MYH9 and MCM2. USP15 is a member of the deubiquitinase family that reverses protein ubiquitination and plays an important regulatory role in various cellular processes as well as in tumour progression.^47^ Therefore, we hypothesised that MYH9 recruits USP15 to enhance the deubiquitination of MCM2, thereby increasing its protein stability. Subsequent verification of this hypothesis indicated that the recruitment of USP15 by MYH9 reduces K48‐linked polyubiquitination of MCM2 and significantly upregulates MCM2 protein levels. Notably, site‐directed mutagenesis assays identified K469 of MCM2 as the key residue for MYH9‐mediated deubiquitination and protein stabilisation.

To further clarify the mechanism through which MCM2 affects CC cell proliferation, a potential interaction between MCM2 and PCNA was explored using the BioGRID database. PCNA functions as a crucial mediator in DNA replication and cell cycle transition and is involved in cell proliferation in various cancers. Studies have shown that PCNA forms complexes with multiple cyclins, thereby regulating cell cycle progression.^48^, ^49^ Here, we showed that MCM2 interacts with PCNA and that the complex formed by these proteins participates in the proliferation of tumour cells.

Finally, the reintroduction of MYH9 in KIF23 KO CC cells partially restored their proliferative ability and DDP resistance. In nude mouse xenograft models, we observed that KIF23 overexpression significantly reduced the sensitivity of CC cells to DDP, and this phenotype was significantly reversed by MYH9 knockdown, accompanied by the downregulation of MCM2 expression.

## 5 CONCLUSIONS

In summary, our results suggest that DDP induces the upregulation of KIF23 expression in a concentration‐ and time‐dependent manner. Acting as a molecular scaffold, KIF23 associates with the deubiquitinase USP7, leading to the K48‐linked deubiquitination of MYH9, resulting in its stabilisation and nuclear translocation. Subsequently, USP15 is recruited to the complex by MYH9 and targets the K469 residue of MCM2, leading to the removal of K48‐linked ubiquitin chains and an increase in MCM2 protein levels. The stabilised MCM2 binds directly to PCNA, and this interaction is associated with the G1/S phase transition and accelerated CC cell proliferation. KIF23 induces CC cell proliferation and DDP chemoresistance by activating the MYH9/MCM2/PCNA signalling pathway, highlighting its potential as a therapeutic target in CC.

## AUTHOR CONTRIBUTIONS

Ying Zhu, Qian Wang, Xiaojie Deng, and Suiqun Guo were responsible for experimental operations and the overall management of the study. Ying Zhu, Yahui Liu, Yilin Zhang, and Haini Fu were responsible for sample collection. Ying Zhu, Qian Wang, Zike Yang, Xiaojie Deng, and Suiqun Guo participated in the experimental design. Ying Zhu, Qian Wang, Yahui Liu, and Yilin Zhang performed data analysis and wrote the manuscript. Zike Yang, Xiaojie Deng, and Suiqun Guo critically revised the manuscript. All authors read and approved the final version of the manuscript.

## CONFLICT OF INTEREST STATEMENT

The authors declare they have no conflicts of interest.

## ETHICS STATEMENT

The Ethics Committee of Southern Medical University approved the experimental and research protocols of this study (approval number: 2025‐ER‐049). All procedures performed in this study were carried out in accordance with the ethical standards of the Institutional Research Committee, as well as the 1964 Declaration of Helsinki and its later amendments and similar ethical standards. Written informed consent was obtained from all patients prior to sample collection. All animal experiments were conducted in strict accordance with the recommendations in the ‘Guide for the Care and Use of Experimental Animals’ of Southern Medical University.

## Supporting information



Supporting Information

## Data Availability

The data supporting the findings of this study are available from the corresponding author upon reasonable request.
