# Supplementary material for: Targeting KIF23 inhibits cell proliferation and primary chemoresistance in cervical cancer by inactivating the MYH9/MCM2/PCNA pathway
Source: Clin Transl Med. 2026 Apr 6;16(4):e70652. doi: 10.1002/ctm2.70652 (PMC13052040; doi:10.1002/ctm2.70652)
Supplement: Supplementary file 1 — Supporting Information [file CTM2-16-e70652-s001.docx]

Supplemental information

**Targeting KIF23 inhibits cell proliferation and primary chemoresistance in cervical cancer by inactivating the MYH9/MCM2/PCNA pathway**

Ying Zhu^1^, Qian Wang^1^, Yilin Zhang^1^, Yahui Liu^2^, Haini Fu^1^, Zike Yang^3*^, Xiaojie Deng^4*^ and Suiqun Guo^1*^

This file includes:

Fig. S1 to S5

Table S1 to S4


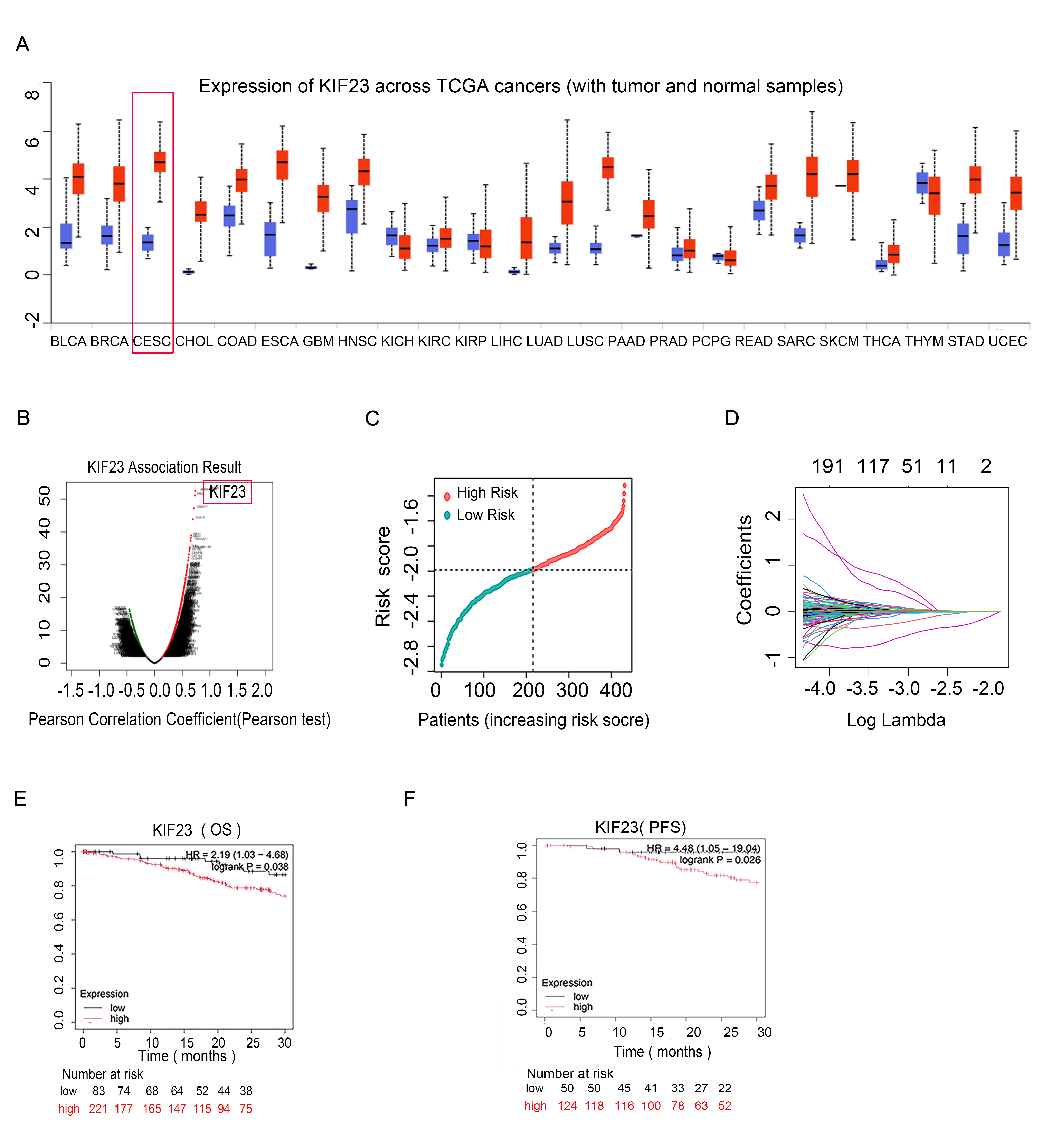


Fig. S1 *KIF23* gene expression and prognosis in CC. (A) Pan-cancer analysis of *KIF23* expression. (B) Volcano plot of genes associated with high *KIF23* expression. (C, D) LASSO risk score plot and LASSO coefficient pathway plot. (E, F) OS and PFS curves for KIF23.
**Abbreviations:** CC, cervical cancer; LASSO, least absolute shrinkage and selection operator; OS, overall survival; PFS, progression-free survival.


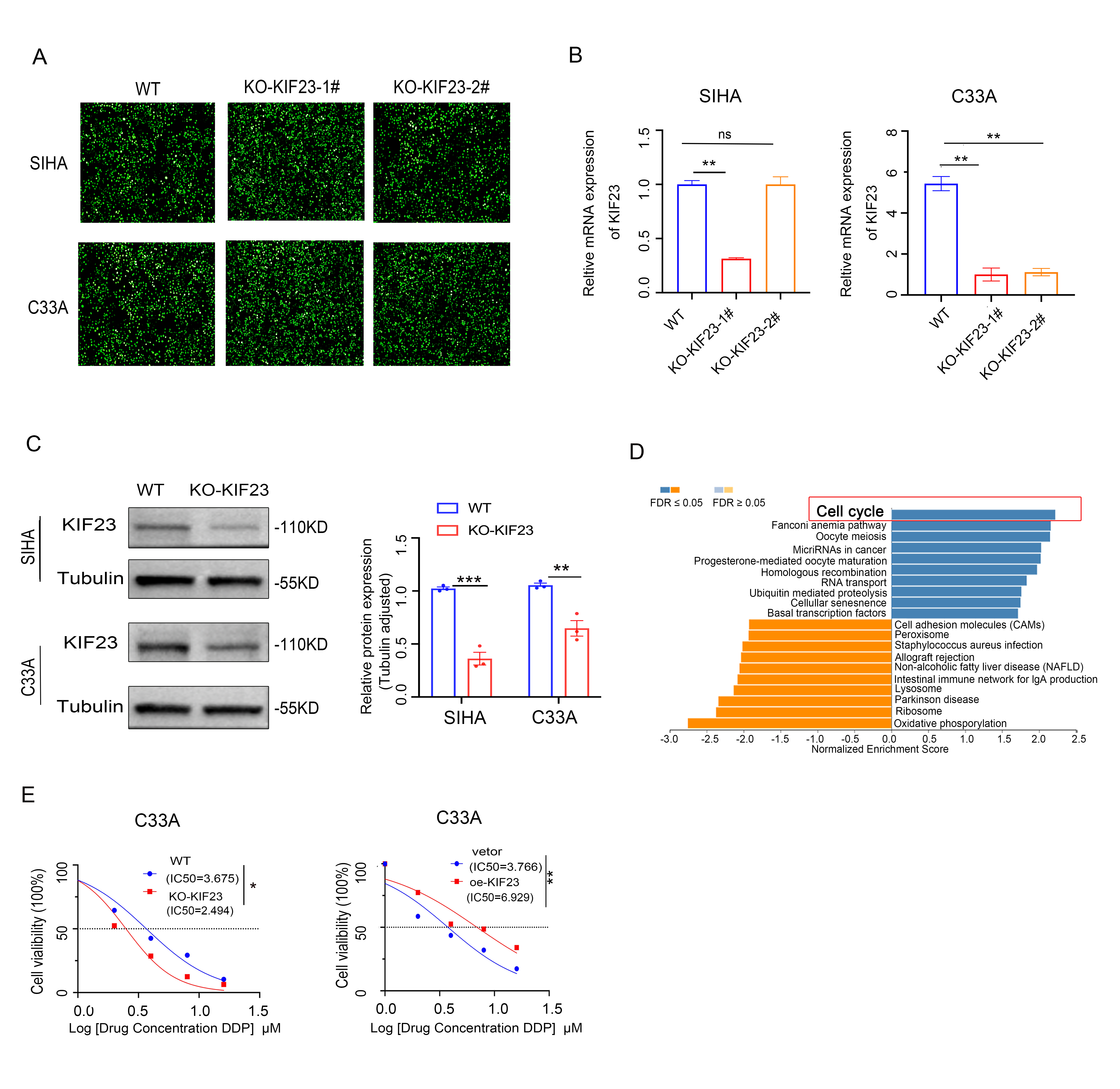


Fig. S2 Knockout of KIF23 inhibits proliferation and regulates the cell cycle in CC cells. (A) Fluorescence imaging showing the transfection efficiency of *KIF23* KO constructs. (B, C) RT-qPCR and WB analyses confirming *KIF23* KO. (D) KEGG pathway enrichment analysis revealing that differentially expressed genes are mainly enriched in the cell cycle pathway. (E) CCK-8 assay showing that *KIF23* KO significantly inhibits cell viability.
**Abbreviations:** CC, cervical cancer; KO, knockout; RT-qPCR, reverse transcription-quantitative polymerase chain reaction; WB, western blotting; KEGG, Kyoto Encyclopedia of Genes and Genomes; CCK-8, Cell Counting Kit-8.


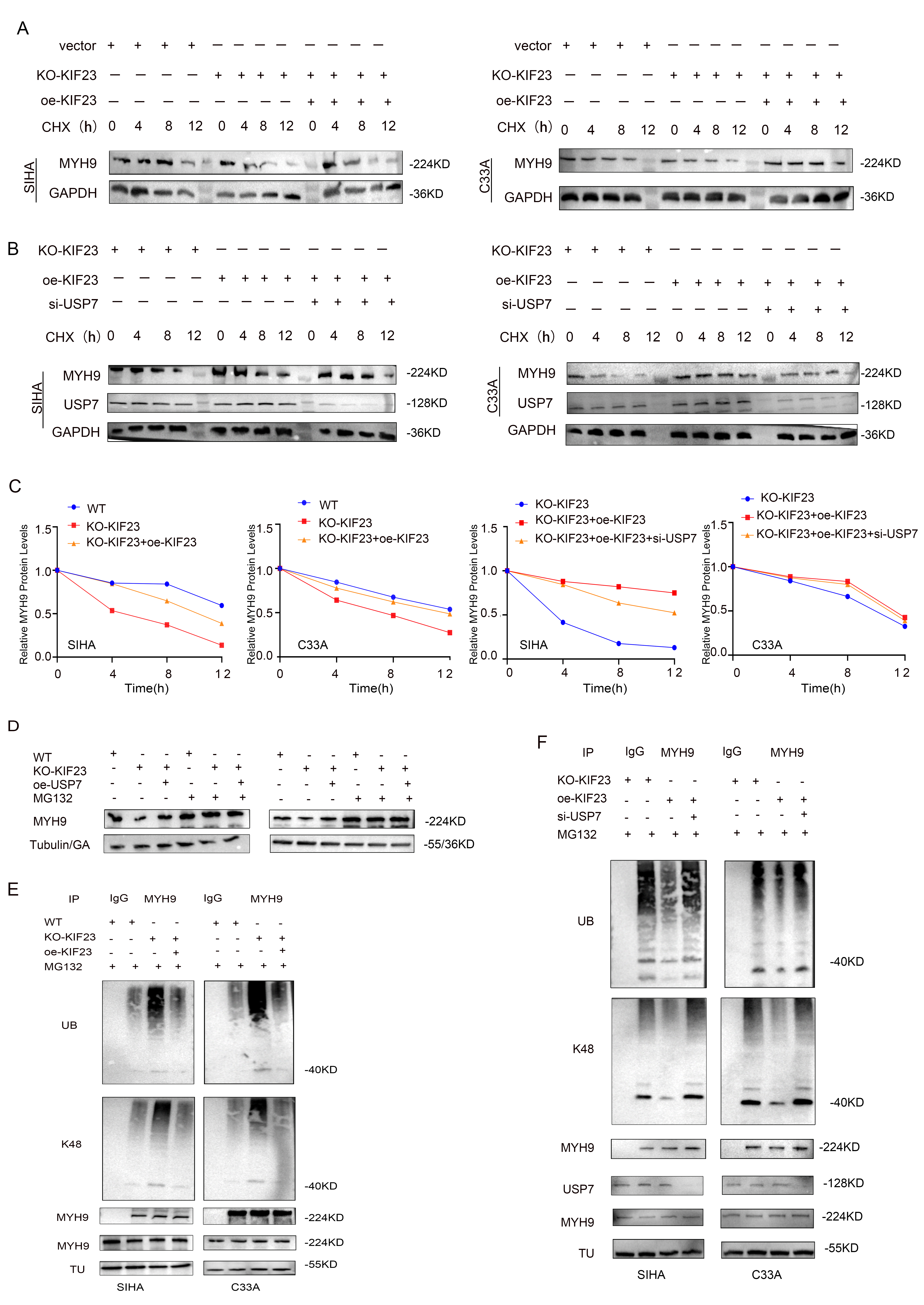


Fig. S3 KIF23 promotes K48-linked polyubiquitination of MYH9 by counteracting USP7-mediated deubiquitination. (A) CHX chase assay determining the half-life of MYH9 protein in *KIF23* KO (KO-*KIF23*), *KIF23* KO with *KIF23* rescue (KO-*KIF23* + oe-*KIF23*), and corresponding control groups in SIHA and C33A cells. (B) CHX chase analysis assessing the effect of USP7 knockdown (si-*USP7*) on MYH9 protein stability in control, KO-*KIF23*, and KO-*KIF23* + oe-*KIF23* SIHA and C33A cells. (C) WB analysis of MYH9 protein levels in WT, KO-*KIF23*, USP7-overexpressing (oe-*USP7*), and MG132-treated groups, confirming the regulatory role of the proteasome pathway. (D) Co-IP and WB analyses detecting MYH9 ubiquitination levels in WT, KO-*KIF23*, *KIF23*-overexpressing (oe-*KIF23*), and MG132-treated SIHA and C33A cells. (E) Co-IP and WB analyses assessing the effects of oe-*KIF23* and oe-*KIF23* combined with USP7 knockdown (oe-*KIF23*+si-*USP7*) on K48-linked polyubiquitination of MYH9 in CC cells treated with MG132.
**Abbreviations:** CHX, cycloheximide; KO, knockout; oe, overexpression; si, small interfering RNA; WB, western blotting; WT, wild-type; Co-IP, co-immunoprecipitation; CC, cervical cancer.


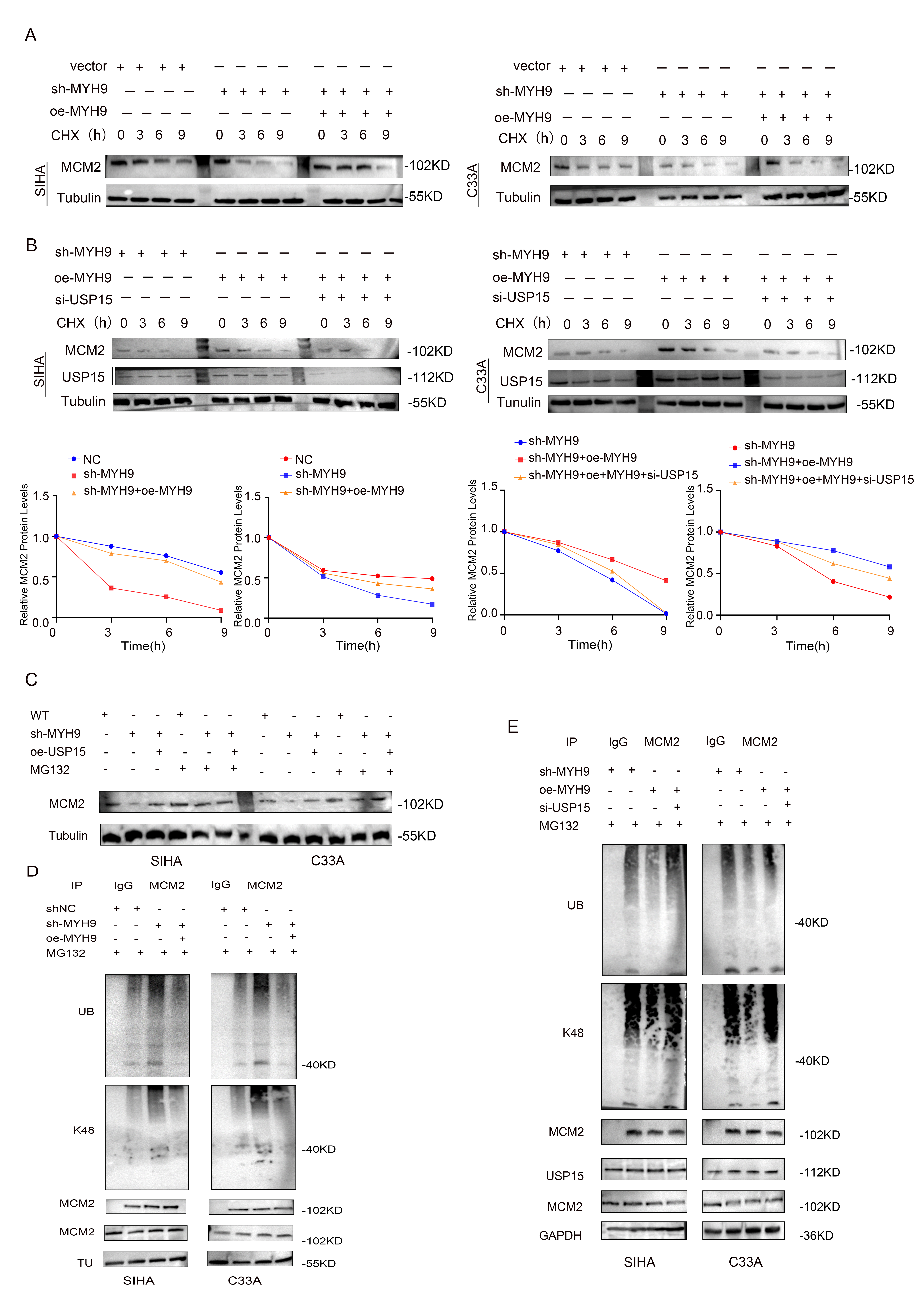


Fig. S4 MYH9 attenuates K48-linked polyubiquitination of MCM2 through recruitment of USP15. (A) CHX chase assay determining the half-life of MCM2 protein in *MYH9* knockdown (sh-*MYH9*), sh-*MYH9* with *MYH9* rescue (sh-*MYH9*+oe-*MYH9*), and corresponding control groups in CC cells. (B) CHX chase analysis assessing the impact of USP15 knockdown (si-*USP15*) on MCM2 protein stability in sh-*MYH9*, sh-*MYH9*+oe-*MYH9*, and control CC cells. (C) WB analysis of MCM2 protein levels in WT, sh-*MYH9*, USP15-overexpressing (oe-*USP15*), and MG132-treated groups, confirming the regulatory role of the proteasome pathway. (D) Co-IP and WB analyses detecting MCM2 ubiquitination levels in WT, sh-*MYH9*, *MYH9*-overexpressing (oe-*MYH9*), and MG132-treated CC cells. (E) Co-IP and WB analyses evaluating the effect of oe-*MYH9*, si-*USP15*, and MG132 treatment on K48-linked polyubiquitination of MCM2 in CC cells.
**Abbreviations:** CHX, cycloheximide; sh, short hairpin RNA; oe, overexpression; CC, cervical cancer; si, small interfering RNA; WB, western blotting; WT, wild-type; Co-IP, co-immunoprecipitation.


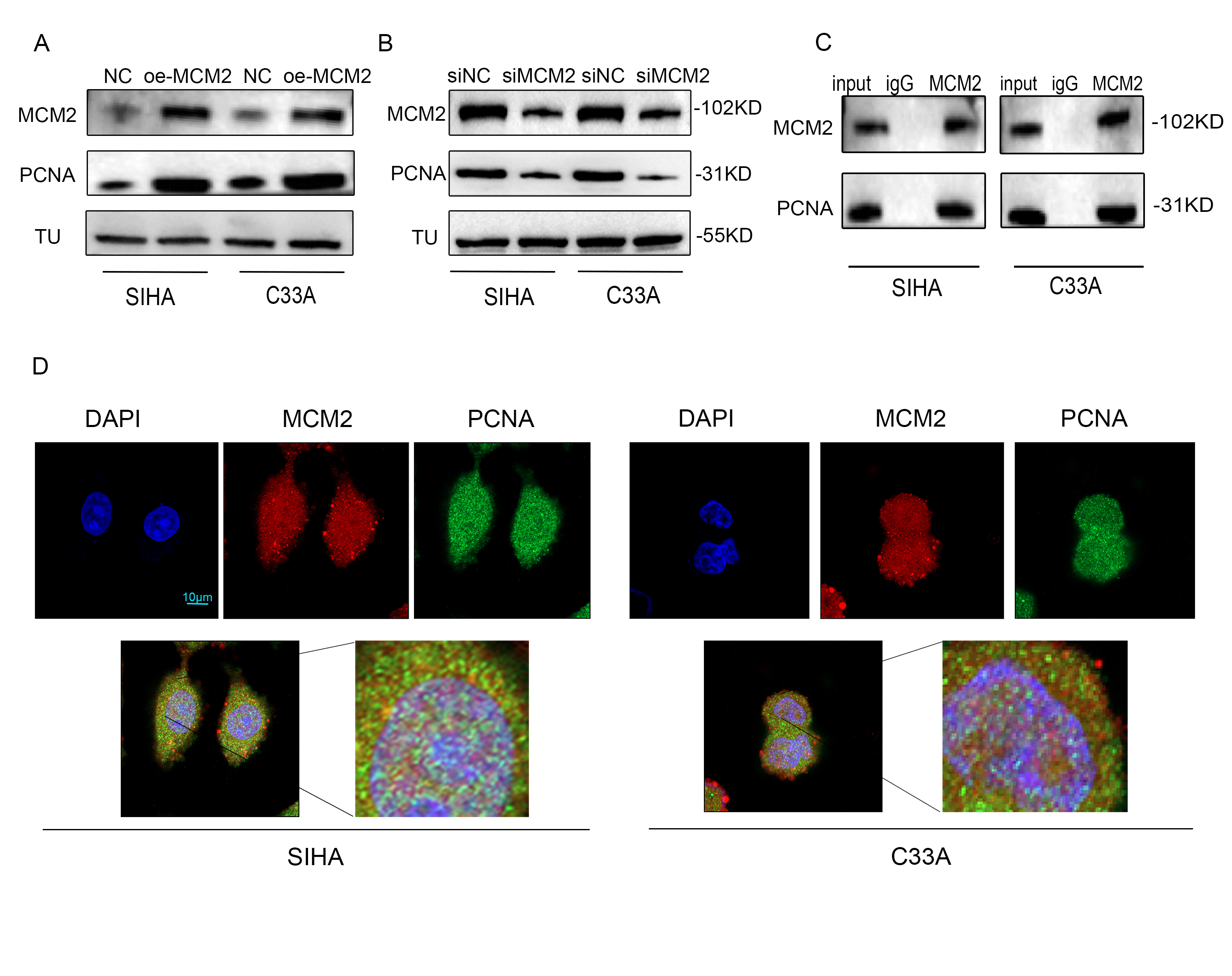


Fig. S5 Interaction between MCM2 and PCNA. (A) WB analysis of PCNA protein levels following MCM2 overexpression (oe-*MCM2*). (B) WB analysis of PCNA protein levels following *MCM2* knockdown. (C) Co-IP analysis detecting the interaction between MCM2 and PCNA in CC cells. (D) Representative confocal microscopy images showing the co-localization of MCM2 and PCNA. Scale bar: 10 µm.
**Abbreviations:** WB, western blotting; oe, overexpression; Co-IP, co-immunoprecipitation; CC, cervical cancer; PCNA, proliferating cell nuclear antigen.

Table S1 The sequences used in this study

| **Accession** | **-10lgP** | **Area** | **Peptides** | **Description** |
| --- | --- | --- | --- | --- |
| sp\|P23246\|SFPQ_HUMAN | 242.67 | 3.14E+08 | 27 | Splicing factor proline- and glutamine-rich |
| sp\|P08670\|VIME_HUMAN | 240.02 | 1.93E+08 | 26 | Vimentin |
| sp\|P11940\|PABP1_HUMAN | 217.58 | 8.81E+07 | 17 | Polyadenylate-binding protein 1 |
| sp\|IQ15233\|NONO_HUMAN | 210.28 | 1.06E+08 | 23 | Non-POU domain-containing octamer-binding 1 |
| sp\|Q9UHB6\|LIMA1_HUMAN | 190.94 | 1.16E+08 | 17 | LIM domain and actin-binding protein 1 |
| sp\|Q96PK6\|RBM14_HUMAN | 185.44 | 2.34E+07 | 11 | RNA-binding protein 14 |
| sp\|P0CG39\|POTEJ_HUMAN | 180.89 | 7.97E+06 | 14 | PTOE ankyrin domain family member J |

| **Name** | **Sequence** |
| --- | --- |
| Homo-KIF23-sg1 | GAACACGCTATGGGAACGGC |
| Homo-KIF23-sg2 | GTCACGAAGCAATTTAGATTG |
| genOFFTM st-h-MCM2_001 | GTCGCAGTTTCTCAAGTAT |
| genOFFTM st-h-MCM2_002 | CTGTCATCCTAGCCAACCA |
| genOFFTM st-h-MCM2_003 | CAGAGCAGGTGACATATCA |

| MYH9 shRNA | Sense | 5’CGCGTCCCCGCAAACCTCGAGAAGGCAATTCAAGAGATTGCCTTCTCGAGGTTTGCTTTTTGGAAAT 3’ |
| --- | --- | --- |
|  | Antisense | 5’CGATTTCCGCAAACCTCGAGAAGGCAATCTCTTGAATTGCCTTCTCGAGGTTTGCGGGGA 3’ |

**Table S2** The primers used in this study

| **Primers name** |  | **Sequence(5’-3’)** |
| --- | --- | --- |
| KIF23 | Forward | TGTGGCTAATCCCTTGGTCAA |
|  | Reverse | AGAACCAGTCATTGTGTGAGTTT |
| MYH9 | Forward | AGTTTGTCTCGGAGCTGTGG |
|  | Reverse | GGTTCGTGTTCCTCAGCGTA |
| GAPDH | Forward | CATGGGTGTGAACCATGAGA |
|  | Reverse | GTCTTCTGGGTGGCAGTGAT |

**Table S3** A list of Antibodies used for WB, IF and IHC

| **Antibody** | **Cat.No** | **Company** | **Species** | **Dulution** |
| --- | --- | --- | --- | --- |
| KIF23 | 28587-1-AP | Proteintech | Rabbit | 1:1000(WB); 1:200(IF); 1:50(IHC) |
| MYH9 | 11128-1-AP | Proteintech | Rabbit | 1:5000(WB); 1:200(IF) |
| MYH9 | 60233-1-Ig | Proteintech | Mouse | 1:2000(WB); 1:50(IF) |
| USP7 | 66514-1-Ig | Proteintech | Mouse | 1:5000(WB); 1:50(IF) |
| CCND1 | 60186-1-Ig | Proteintech | Mouse | 1:1000(WB) |
| P21 | 10355-1-AP | Proteintech | Rabbit | 1:2000(WB) |
| β-tublin | 80762-1-RR | Proteintech | Rabbit | 1:5000(WB) |
| PCNA | 10205-2-AP | Proteintech | Rabbit | 1:1500(IHC) |
| Ki-67 | 27309-1-AP | Proteintech | Rabbit | 1:2000(IHC) |
| USP15 | 14354-1-AP | Proteintech | Mouse | 1:2000(WB) |
| MCM2 | 10513-1-AP | Proteintech | Rabbit | 1:1000(WB) |
| Histone3 | 17168-1-AP | Proteintech | Rabbit | 1:2000(WB) |
| ubiquitin | PAb 10201-2-AP | Proteintech | Rabbit | 1:1000(WB) |
| K48 | mAb #8081 | Cell Sigaling | Rabbit | 1:1000(WB) |
| HA tag | 51064-2-AP | Proteintech | Rabbit | 1:5000(WB) |
| Beta Actin | 66009-1-Ig | Proteintech | Mouse | 1:5000(WB) |
| MYC tag | 16286 - 1 -AP | Proteintech | Rabbit | 1:5000(WB) |
| Flag-tag | F1804 | Sigma | Mouse | 1:1000(WB) |
| His-tag | 66005-1-Ig | Proteintech | Mouse | 1:5000(WB) |
| β-actin | 20536-1-AP | Proteintech | Rabbit | 1:5000(WB) |

**Table S4** Correlation between KIF23 protein expression and clinical pathological parameters

| Parameters | n | Expression of KIF23 | | p-value |
| --- | --- | --- | --- | --- |
|  |  | Low expression | High expression |  |
| Age | | | | 0.1130 |
| ≥55 years | 30 | 6 | 24 |  |
| <55 years | 93 | 33 | 60 |  |
| Clinical stage | | | | 0.0334 |
| I+II | 19 | 9 | 10 |  |
| III+IV | 86 | 20 | 66 |  |
| T stage | | | | 0.3379 |
| T1/T2 | 83 | 24 | 59 |  |
| T3 | 40 | 15 | 25 |  |
| N stage | | | | 0.6041 |
| N0 | 95 | 29 | 66 |  |
| N1 | 28 | 10 | 18 |  |
| M stage | | | | 0.5253 |
| M0 | 114 | 37 | 77 |  |
| M1 | 9 | 2 | 7 |  |
| Vital status | | | | 0.8487 |
| Alive | 99 | 31 | 68 |  |
| Dead | 24 | 8 | 16 |  |

KIF23, kinesin family member 23

**Table S5** Univariate and multivariate Cox regression analysis of individual parameters correlated with overall survival in patients with CC

| Characteristics | Univariate Cox | | | Multivariate Cox | | |
| --- | --- | --- | --- | --- | --- | --- |
|  | Hazard ratio | 95% CI | P-value | Hazard ratio | 95% CI | P-value |
| Age (<55 vs. ≥ 55) | 1.123 | 0.587-2.151 | 0.732 |  |  |  |
| T stage (T1-2 vs. T3-4) | 1.872 | 0.945-3.709 | 0.072 |  |  |  |
| N stage (N0 vs. N1) | 1.935 | 0.981-3.816 | 0.057 |  |  |  |
| Clinical stage (I-II vs. III-IV) | 2.156 | 1.089-4.269 | 0.028 | 2.215 | 1.092-4.492 | 0.028 |
| KIF23 Expression (Low vs. High) | 2.842 | 1.417-5.702 | 0.003 | 2.619 | 1.287-5.331 | 0.008 |
